# Aporphine and isoquinoline derivatives block glioblastoma cell stemness and enhance temozolomide cytotoxicity

**DOI:** 10.1038/s41598-022-25534-2

**Published:** 2022-12-07

**Authors:** Dorival Mendes Rodrigues-Junior, Cristiano Raminelli, Haifa Hassanie, Gustavo Henrique Goulart Trossini, Givago Prado Perecim, Laia Caja, Aristidis Moustakas, André Luiz Vettore

**Affiliations:** 1 Department of Medical Biochemistry and Microbiology, Science for Life Laboratory, Biomedical Center, Uppsala University, Uppsala, Sweden; 2 Departamento de Química, Universidade Federal de São Paulo, Campus Diadema, Diadema, Brazil; 3 Departamento de Ciências Biológicas, Universidade Federal de São Paulo, Campus Diadema, Diadema, Brazil; 4 Departamento de Farmácia, Cidade Universitária, Universidade de São Paulo, São Paulo, Brazil

**Keywords:** Cancer, Drug discovery, Neuroscience, Cell biology, Cell death, Cancer stem cells

## Abstract

Glioblastoma (GBM) is the most aggressive and common primary malignant brain tumor with limited available therapeutic approaches. Despite improvements in therapeutic options for GBM patients, efforts to develop new successful strategies remain as major unmet medical needs. Based on the cytotoxic properties of aporphine compounds, we evaluated the biological effect of 12 compounds obtained through total synthesis of ( ±)-apomorphine hydrochloride (APO) against GBM cells. The compounds 2,2,2-trifluoro-1-(1-methylene-3,4-dihydroisoquinolin-2(1*H*)-yl)ethenone (A5) and ( ±)-1-(10,11-dimethoxy-6a,7-dihydro-4*H*-dibenzo[*de*,*g*]quinolin-6(5*H*)-yl)ethenone (C1) reduced the viability of GBM cells, with 50% inhibitory concentration ranging from 18 to 48 μM in patient‐derived GBM cultures. Our data show that APO, A5 or C1 modulate the expression of DNA damage and apoptotic markers, impair 3D‐gliomasphere growth and reduce the expression of stemness markers. Potential activity and protein targets of A5, C1 or APO were predicted in silico based on PASS and SEA software. Dopamine receptors (DRD1 and 5), CYP2B6, CYP2C9 and ABCB1, whose transcripts were differentially expressed in the GBM cells, were among the potential A5 or C1 target proteins. Docking analyses (HQSAR and 3D-QSAR) were performed to characterize possible interactions of ABCB1 and CYP2C9 with the compounds. Notably, A5 or C1 treatment, but not temozolomide (TMZ), reduced significantly the levels of extracellular ATP, suggesting ABCB1 negative regulation, which was correlated with stronger cytotoxicity induced by the combination of TMZ with A5 or C1 on GBM cells. Hence, our data reveal a potential therapeutic application of A5 and C1 as cytotoxic agents against GBM cells and predicted molecular networks that can be further exploited to characterize the pharmacological effects of these isoquinoline-containing substances.

## Introduction

Glioblastoma (GBM) is a brain malignancy considered one of the most lethal human tumors, with few long-term survivors, which affects mainly adults and exhibits a high degree of inflammation in its microenvironment, of infiltration and acquired resistance to chemo- or radio-therapy^1,2^. GBMs are known for their progenitor-like and self-renewing stem-like cells, which have been demonstrated to generate the ability to resist various treatments^3^. Such glioma stem-like cells, by means of their resistance phenotype, contribute to the formation of recurrent, post-operative tumors^4^. Transcriptional profiling is used to classify GBM into proneural (PN), classical (CL) and mesenchymal (MS) subtypes^5^. The current standard of care for newly diagnosed GBM patients is surgical tumor resection followed by postoperative chemotherapy with temozolomide (TMZ), radiation therapy (RT), and then nitrosoureas (such as lomustine or vincristine) as second choices after adjuvant the TMZ-based chemotherapy^1–7^. Although aggressive multimodal therapy has improved patient survival, high-grade GBM is associated with a poor prognosis and resistance to initial treatments inevitably leading to tumor regrowth/recurrence, providing an unfavorable endpoint of the established anti-tumor approaches^1,7^ Hence, the discovery of new potential agents to control GBM growth may contribute to meeting the urgent and unmet clinical need of improving patient survival.

The alkaloids represent one of the most well-known classes of natural products with multiple pharmacological properties^8^. Some aporphine alkaloids isolated from plants or obtained by semi- or total synthesis displayed activities related to anticonvulsant, antiplatelet aggregation, anti-HIV, dopaminergic, antispasmodic and anticancer effects^9–13^. Among aporphine alkaloids derivatives, apomorphine is a potent dopamine D1/D2 receptor agonist largely used for the treatment of Parkinson's disease^14–16^ with potential anti-proliferative activity against different tumors^17–20^, including GBM^21,22^. In more detail, Pinheiro et al.^22^ reported that (*R*)-(-)-APO reduced the viability of GBM patient-derived cells (U3005MG, U3028MG and U3046MG), with IC_50_ ranging from 29.01 to 38.68 µM. In addition, Lee et al.^21^ observed 10 µM APO reducing significantly the growth of GBM cells. Furthermore, apomorphine was reported to induce cytotoxicity at high doses (100 to 400 µM) in rat glioma C6 cells through necrotic cell death^23^.

We previously showed that the compounds 2,2,2-trifluoro-1-(1-methylene-3,4-dihydroisoquinolin-2(1*H*)-yl)ethanone (A5*;* i.e. an isoquinoline derivative) and ( ±)-1-(10,11-dimethoxy-6a,7-dihydro-4*H*-dibenzo[*de*,*g*]quinolin-6(5*H*)-yl)ethanone (C1*;* i.e. an aporphine derivative), which were acquired as intermediates of convergent total synthesis of ( ±)-apomorphine hydrochloride (APO), presented cytotoxicity against head-and-neck squamous cell carcinoma (HNSCC)^24^. Hence, the present study aimed to evaluate the cytotoxic effects of 12 compounds produced through the total synthesis of APO from 2-phenethylamine and 3,4-dimethoxybenzaldehyde, individually or in combination with TMZ treatment, on GBM cells, as well as, their impact on cell death and GBM stem cell (GSC) formation.

## Results

### Antitumor effects of aporphine- and isoquinoline-derivatives in GBM cell lines

**Figure 1:**
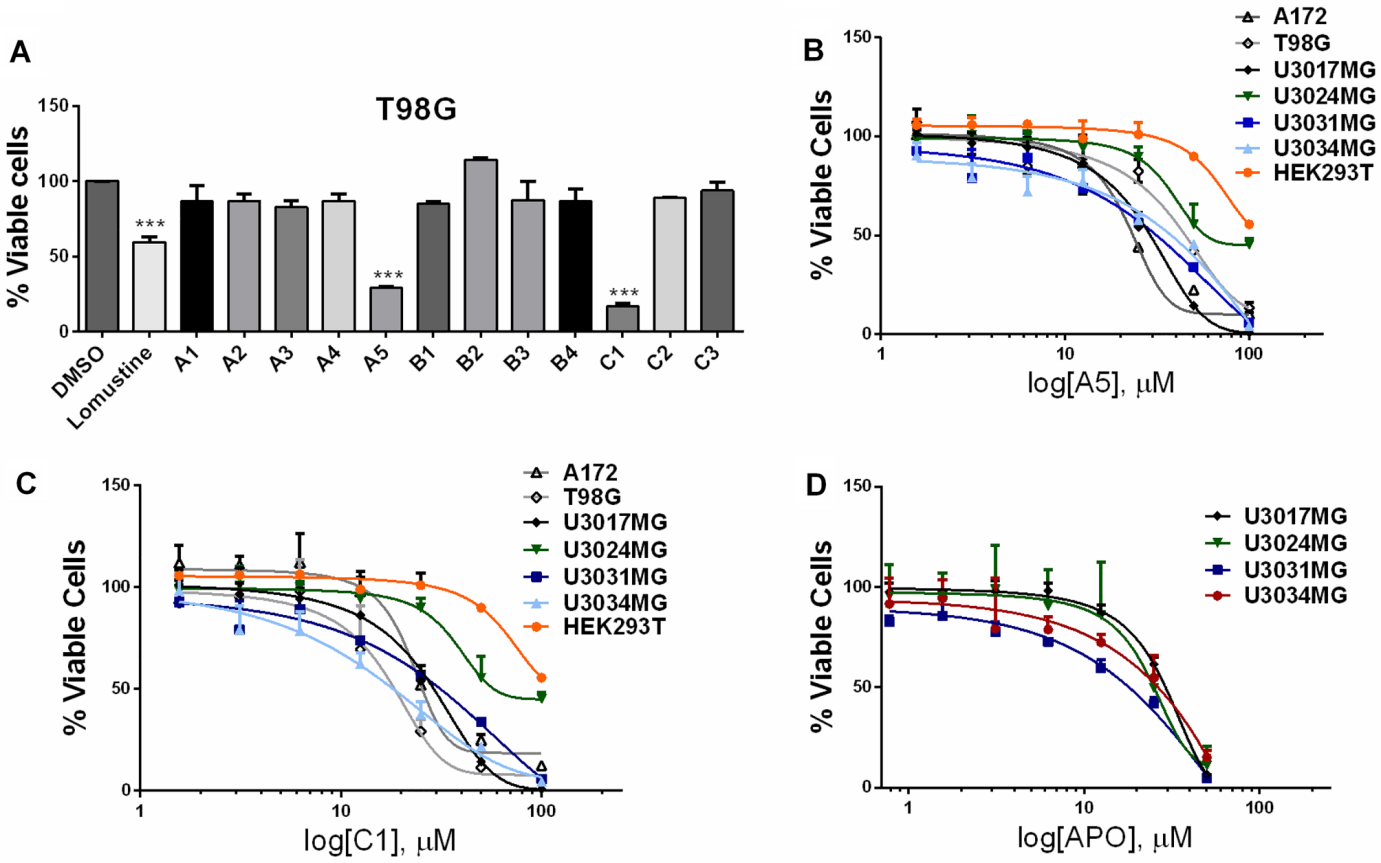
Assessment of cell viability in GBM cells. (**A**) Viability level of T98G cells after treatment for 48 h with 100 µM of selected substances tested in this study, as well as 12.5 µM of lomustine, used as a positive control for GBM cytotoxicity. Data were normalized against DMSO. IC_50_ values were determined after treatment for 48 h with increasing concentrations of A5 (**B**), C1 (**C**) or APO (**D**). Cell viability was determined using the PrestoBlue reagent. These assays were conducted in triplicate (with at least two biological replicates); error bars represent mean ± SD. Asterisks denote significance as determined by one-way ANOVA multiple comparisons followed by Dunnet test (post-test); ***p < 0.0001.

The in vitro antitumor activities of 12 (hetero)aromatic compounds obtained through convergent total syntheses of APO^25^ (A1, A2, A3, A4, A5, B1, B2, B3, B4, C1, C2 and C3, with chemical structures presented in Suppl. Figure S1A) were evaluated on the GBM T98G (ATCC; CRL-1690) cell line. The treatment of T98G cells for 48 h with 100 μM of the (hetero)aromatic compounds showed a significant reduction in their cell viability promoted by A5 and C1, while A1, A2, A3, A4, B1, B2, B3, B4, C2 and C3 did not affect T98G cell viability (Fig. 1A). Lomustine was used as a positive control of cytotoxicity (Fig. 1A). Furthermore, in silico prediction analysis (http://www.cbligand.org/BBB) showed that A5 and C1, as well as APO, were expected to pass through the brain-blood barrier (BBB) with positive scores: 0.142, 0.127 and 0.092, respectively (Suppl. Figure S1B). In tandem, these data indicate that A5 and C1 substances could have antitumor activity against GBM cells.

Since A5 and C1 presented cytotoxic effects against T98G cells, a 48 h treatment with increasing concentrations of these compounds was conducted in the commercially available GBM cells T98G and A172 (ATCC; CRL-1620), in four GBM patient-derived cells from grade IV glioma biopsies (https://www.hgcc.se/) ^26^: U3017MG (CL subtype), U3024MG (MS subtype), U3031MG (MS subtype) and U3034MG (MS subtype) and in a non-tumorigenic embryo kidney cell line HEK293T (ATCC; CRL-3216). These assays revealed a dose-dependent reduction of cell viability promoted by A5 and C1 in all GBM cells assessed (Fig. 1B and C). Patient-derived GBM cells treated with increasing concentrations of APO also showed a similar dose-dependent cytotoxic response (Fig. 1D). The IC_50_ values for A5 on GBM cell lines ranged from 23.26 to 48.36 μM, while the IC_50_ concentration for C5 was between 16.91 and 30.97 μM. In the same range, the IC_50_ values for APO on patient-derived GBM cells varied from 20.51 to 28.02 μM (Table 1). On the other hand, HEK293T cells were more resistant to A5 and C1 in comparison to the GBM cells (Fig. 1B and C) with IC_50_ concentrations of 63.51 and 68.63 μM for A5 and C1, respectively (Table 1). Dose-dependent response to TMZ on the patient-derived GBM cells was likewise performed based on cell viability (Suppl. Figure S2).

**Table 1:** Chemical structures and IC_50_ values of A5, C1 and APO on the cell lines.

Compound structure	Cell Line IC_50_ µM						
	U3017MG	U3024MG	U3031MG	U3034MG	A172	T98G	HEK293T
	27.05 ± 1.91	48.36 ± 4.54	39.02 ± 6.54	26.95 ± 3.42	23.26 ± 2.81	32.14 ± 6.54	63.51 ± 4.83
	29.85 ± 2.27	30.97 ± 7.17	24.78 ± 1.49	18.26 ± 2.27	26.15 ± 4.86	16.91 ± 0.73	68.63 ± 6.22
	28.02 ± 5.51	23.95 ± 1.19	20.51 ± 1.59	26.02 ± 5.51	–	–	–

### Cell death induced by aporphine and isoquinoline compounds

**Figure 2:**
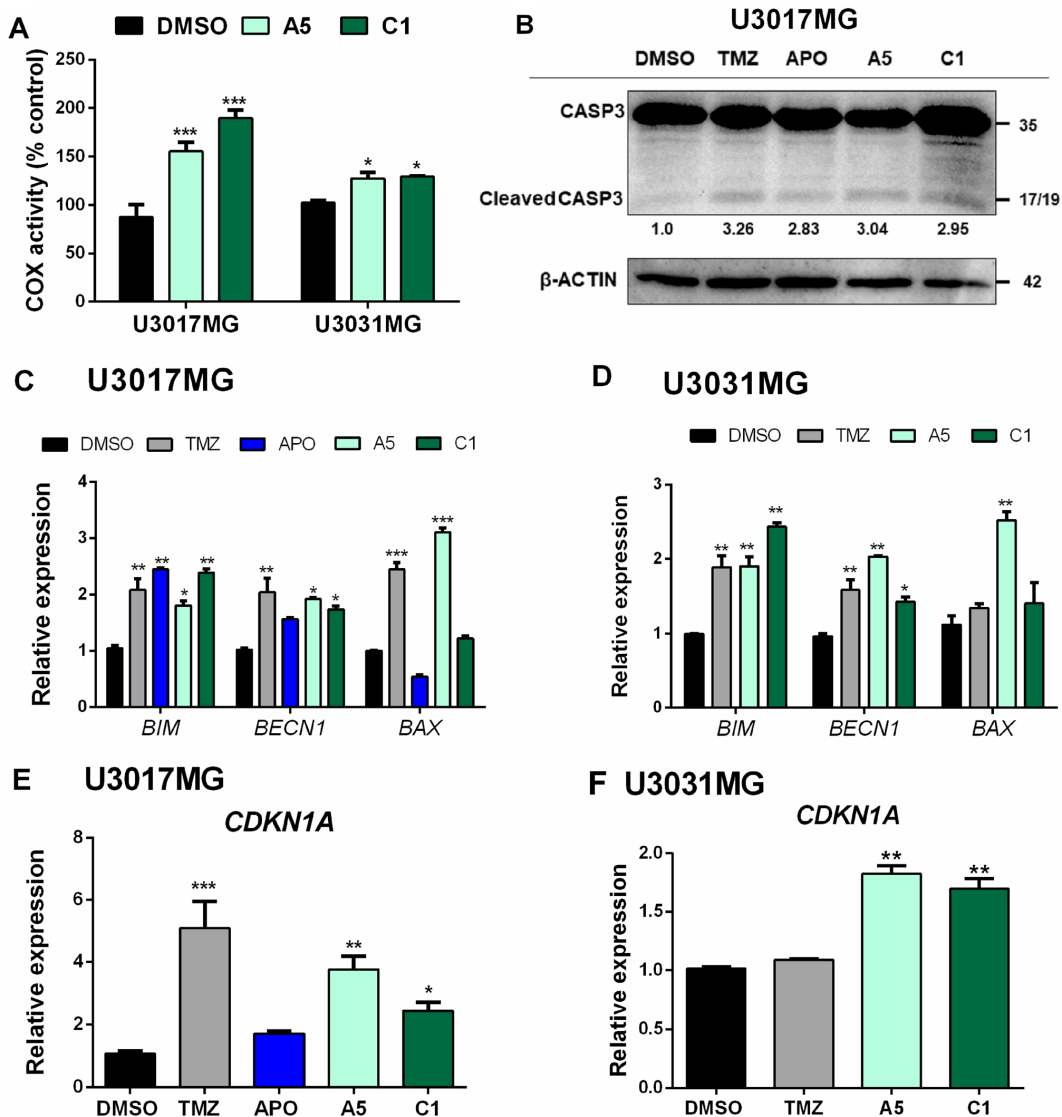
Treatment with aporphine and isoquinoline derivatives induce an intrinsic apoptosis cell death. (**A**) COX activities of GBM cells after 24 h of incubation with A5 (30 μM) or C1 (30 μM). Results are expressed as percentage of cell control treated with DMSO. (**B**) Representative immunoblot of cleaved caspase-3 in U3017MG cells treated with TMZ, APO, A5 or C1 for 48 h. β-Actin was used as a loading control, and molecular mass (kDa) markers are indicated along with densitometric values of normalized band intensity. (**C, D**) RT-qPCR was used for detection of markers for cell death mediated by apoptosis: *BIM*, *BECN1* and *BAX* in U3017MG (**C**) and U3031MG (**D**) cells treated with DMSO, TMZ (150 µM), APO (25 µM), A5 (30 µM) or C1 (30 µM) for 48 h. TMZ, an alkylating agent frequently used as chemotherapy in GBM patients, was used as the positive control. (**E, F**) RT-qPCR was used for detection of DNA damage response gene expression: *CDKN1A* (*p21*) in U3017MG (**E**) and U3031MG (**F**) cells treated with DMSO, TMZ (150 µM), APO (25 µM), A5 (30 µM) or C1 (30 µM) for 48 h. All the relative expression levels were normalized to *GAPDH* expression and calculated using the 2^−ΔΔCt^ method. Error bars represent SD from three biological replicates and *p* values were calculated according to the two-way ANOVA test followed by Bonferroni (post-test); *p < 0.05, **p < 0.01, ***p < 0.001.

In a previous study, we reported that A5, C1 and APO induced DNA damage and cell death in HNSCC cells^24^. Hence, we sought to characterize whether these compounds induced such effects on the patient-derived GBM cells, based on the analysis of cytochrome C oxidase (COX) activity and expression of cell markers for apoptosis and DNA damage response (DDR). First, it was observed that U3017MG and U3031MG cells treated with 30 μM of A5 and C1 for 24 h had a significant increase of COX activity in comparison to DMSO, which suggests a higher release of pro-apoptotic factors from the mitochondria of treated cells (Fig. 2A). Then, to better describe the mechanism driving the cell death induced by these aporphine and isoquinoline derivatives on GBM cells, we evaluated the presence of cleaved caspase-3 in U3017MG cells after 48 h-treatment with 150 μM TMZ, 25 μM APO, 30 μM A5 or 30 μM C1, which corresponds approximately to the IC_50_ value for each compound. The detection of cleaved caspase-3 after treatment with these compounds confirmed that A5 and C1 induced cell death mediated by the intrinsic apoptosis pathway (Fig. 2B), as suggested by the correspondingly higher COX activity (Fig. 2A). These data were further validated by a significant increment in the mRNA levels of Bcl-2-like protein 11 (*BCL2L11*; i.e. *BIM*) and beclin-1 (*BECN1*) in U3017MG and U3031MG cells after treatment with A5 or C1 (Fig. 2C and D). Of note, the expression of the apoptotic regulator BAX (*BAX*) was significantly enhanced in U3017MG and U3031MG cells treated with A5 (Fig. 2C and D).

Additionally, as shown in Fig. 2E, the cyclin-dependent kinase inhibitor 1 (*CDKN1A*; i.e. p21) was significantly upregulated in U3017MG cells at the mRNA levels after treatment with TMZ, A5 or C1, and likewise upregulated in U3031MG cells treated with A5 and C1 (Fig. 2F). Of importance, the G2 cell cycle phase/mitotic-specific cyclin-B1 (*CCNB1*) expression was downregulated after treatment with all four compounds in U3017MG cells (Suppl. Figure S3). Together, these data suggest the activation of p53 in the context of response to DNA damage induced by A5 or C1, which leads to cell death mediated by apoptosis.

### Aporphine and isoquinoline derivatives suppress GBM stem cell formation

**Figure 3:**
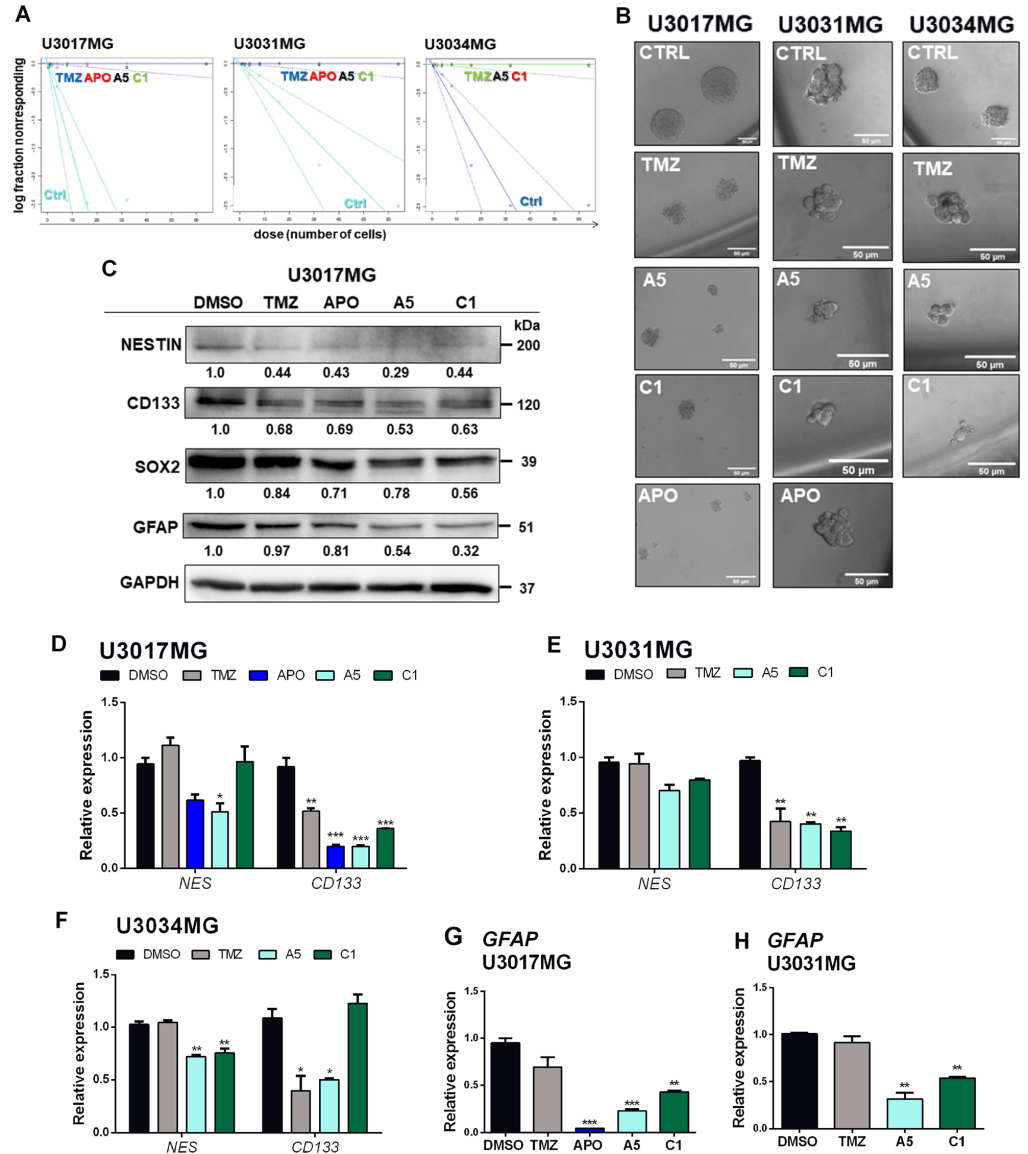
Aporphine and isoquinoline derivatives block gliomasphere formation. (**A**) Extreme limiting dilution assay of three patient-derived GBM cell lines (U3017MG, U3031MG and U3034MG) treated with DMSO (CTRL), TMZ (150 µM), APO (25 µM), A5 (30 µM) or C1 (30 µM) for seven days. The number of sphere‐containing wells (> 50 μm) per plating density was plotted. Steeper slopes indicate higher frequencies of sphere‐forming cells. (**B**) Representative microscopy figures of gliomaspheres formed after treatment with CTRL, TMZ, A5, C1 or APO for seven days. Magnification bars: 50 μm. (**C**) Immunoblot analysis of NESTIN (NES), CD133, SOX2, and GFAP levels in U3017MG cells; GAPDH was used as a normalization control for protein analysis, and molecular mass (kDa) markers are indicated along with densitometric values of normalized band intensity. The grouping of blots was cropped from different parts of the same gel. RT-qPCR was used for detection of stemness markers *NES* and *CD133* in U3017MG (**D**), U3031MG (**E**) and U3034MG (**F**) treated with TMZ, APO, A5 or C1 for 48 h. RT-qPCR was used for detection of *GFAP* in U3017MG (**G**) and U3031MG (**H**) treated with TMZ, APO, A5 or C1 for 48 h. All the relative expression levels were normalized to *GAPDH* expression and calculated using the 2^−ΔΔCt^ method. Error bars represent SD from three biological replicates (*p < 0.05, **p < 0.01, ***p < 0.001).

GSCs have been implied in GBM therapeutic resistance^4,27^; we therefore, sought to assess the impact of TMZ, APO, A5 and C1 on gliomasphere formation using the extreme limiting dilution analysis (ELDA)^28^ assay. Gliomasphere formation was observed after culture for seven days in the stem cell medium of the three patient-derived cell lines (U3017MG, U3031MG and U3034MG); however, in the presence of TMZ, APO, A5 and C1, no gliomaspheres with a diameter bigger than 50 μm were observed, as represented by the overlapped curves for TMZ, APO, A5 and C1 in Fig. 3A. Moreover, the cell aggregates formed after these treatments were morphologically different from the respective gliomasphere controls (Fig. 3B).

Of note, the expression of GBM stemness markers, such as NESTIN (*NES*), CD133 (*PROM1*) and SOX2, as well as the astrocytic marker glial fibrillary acidic protein (GFAP), were downregulated in U3017MG cells after 48 h-treatment with TMZ, APO, A5 or C1 (Fig. 3C). In concordance with the protein data, the *CD133* transcript levels were also found significantly diminished after TMZ and A5 treatment in U3017MG, U3031MG and U3034MG cells, and after C1 treatment in U3017MG and U3031MG cells (Fig. 3D–F). Moreover, *NES* expression was significantly reduced only in U3034MG cells exposed to A5 and C1, and in U3017MG cells exposed to A5 (Fig. 3D–F). The data of *NES* expression suggested that these compounds might modulate NESTIN levels via transcriptional or post-transcriptional (via non-coding RNAs for instance) regulatory mechanisms. Additionally, the levels of *GFAP* transcripts were downregulated in U3017MG and U3031MG cells after treatment with A5 and C1 (Fig. 3G and H). By comparing GFAP protein (Fig. 3C) to *GFAP* mRNA (Fig. 3G) levels in the U3017MG cells, it appears as if the potency of APO is stronger at the mRNA than at the corresponding protein level. Yet, this analysis demonstrates the downregulation of GFAP expression at the mRNA and protein levels and whether one of the compounds presents additional post-transcriptional effects on GFAP expression remains to be examined in deeper detail.

### Predictions of pharmacological activity and interacting proteins for aporphine and isoquinoline derivatives

**Figure 4:**
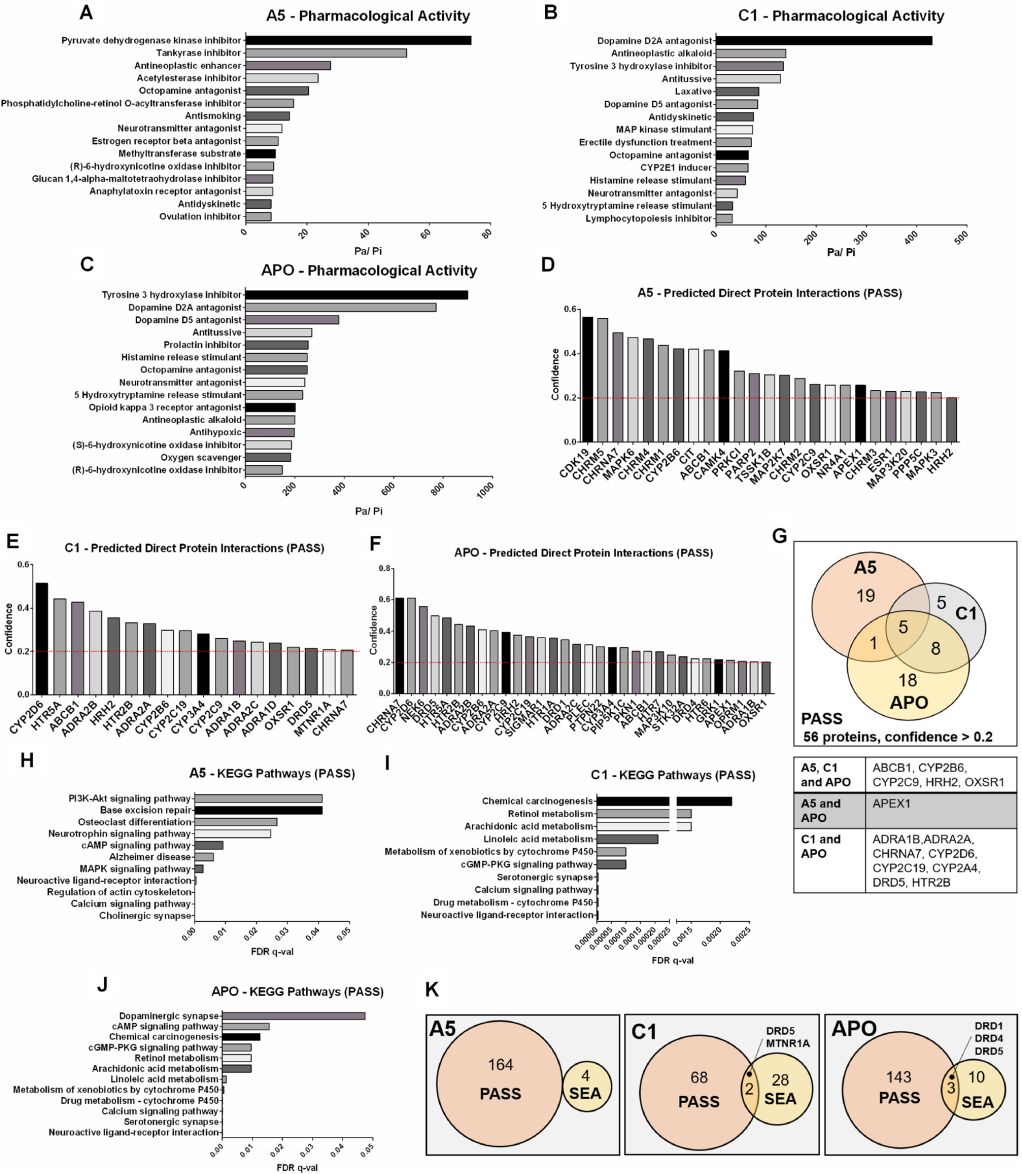
In silico predictions of pharmacological activity and target proteins for the aporphine and isoquinoline derivatives. Prediction of Activity Spectra for Substances (PASS; http://way2drug.com/PassOnline) identified the predicted pharmacological activity of tested compounds (**A**) A5, (**B**) C1 and (**C**) APO. The data were represented as the ratio of probable activity (Pa) per probable inactivity (Pi) for each pharmacological activity. Representation of the PASS predicted proteins with confidence > 0.2 interacting with (**D**) A5, (**E**) C1 or (**F**) APO. (**G**) Distribution of the 56 predicted proteins through PASS (confidence > 0.2) interacting with A5, C1 or APO. The table lists the common interacting proteins for A5, C1 and APO (ABCB1, CYP2B6, CYP2C9, HRH2 and OXSR1); A5 and APO (APEX1); C1 and APO (ADRA1B, ADRA2A, CHRNA7, CYP2D6, CYP2C19, CYP2A4, DRD5 and HTR2B). Representation of the significant KEGG pathways identified through the STRING database related to the PASS predicted proteins interacting with (**H**) A5, (**I**) C1 and (**J**) APO. (**K**) Distribution of the common predicted proteins interacting with A5, C1 and APO based on PASS and Similarity Ensemble Approach (SEA).

In order to provide an insight into the biological activity of the aporphine and isoquinoline investigated in this study, we applied two independent in silico approaches to characterize the biological activity spectrum based on the chemical structure and to predict direct chemical-protein interactions with the A5, C1 and APO compounds. First, using PASS^29^, we predicted a spectrum of pharmacological activity and toxic effects, which is represented by the ratio of calculated probabilities Pa (“active”)/ Pi (“inactive”). Based on this prediction, the compound A5 had a high probability to act as a pyruvate dehydrogenase kinase inhibitor (Pa/Pi = 73.666), as a tankyrase inhibitor (Pa/Pi = 52.5), as an antineoplastic enhancer (Pa/Pi = 27.692) and as an acetylesterase inhibitor (Pa/Pi = 23.666) (Fig. 4A). Likewise, C1 might act as a dopamine D2A antagonist (Pa/Pi = 431.0), antineoplastic alkaloid (Pa/Pi = 139.5), tyrosine 3 hydroxylase inhibitor (Pa/Pi = 134.5), dopamine D5 antagonist (Pa/Pi = 83.25), as well as, a CYP2E1 inducer (Pa/Pi = 64.0) (Fig. 4B). Apart from the effect as antagonist of dopamine D2A (Pa/Pi = 771.0), dopamine D5 (Pa/Pi = 378.5) and inhibitor of tyrosine 3 hydroxylase (Pa/Pi = 899.0), APO was predicted to act as an antineoplastic alkaloid (Pa/Pi = 200.0) (Fig. 4C). Additionally, minor toxic side-effects were predicted for A5 (shivering, neutrophilic dermatosis and withdrawal), C1 (hypothermic, excitability, and yawning) or APO (piloerection, panic and hypothermia) (Suppl. Figure S4).

Due to the high number of predicted proteins interacting with the A5, C1 and APO compounds estimated by the PASS algorithm (predicted proteins for A5: 164; C1: 70; APO: 146), an arbitrary cutoff of > 0.2 was established to proceed with further analysis (Suppl. Figure S5). Thus, among the 25 proteins with a high probability of interaction with A5 were CDK19, CHRM5, MAPK6, CYP2B6, ABCB1 and CYP2C9 (Fig. 4D), while CYP2D6, HTR5A, ABCB1, CYP2B6, CYP2C9 and DRD5 were among the 18 proteins with which C1 could interact (Fig. 4E). Besides, apart from the dopamine receptors DRD5, DRD4 and DRD1, the proteins CHRNA7, CYP2D6, CYP2B6 and ABCB1 were also among the 19 proteins predicted to have direct interaction with APO (Fig. 4F). It does not go unnoticed that according to this analysis the compounds A5, C1 and APO shared five possible common target proteins: ABCB1, CYP2B6, CYP2C9, HRH2 and OXSR1 (Fig. 4G). Along the same line, A5 and APO shared APEX1 as a putative target, while C1 and APO shared eight possible target proteins (ADRA1B, ADRA2A, CHRNA7, CYP2D6, CYP2C19, CYP2A4, DRD5 and HTR2B).

Furthermore, a protein–protein interaction network was acquired using the Search Tool for the Retrieval of Interacting Genes (STRING) database based on the list of predicted target proteins for A5, C1 and APO (Suppl. Figure S6). Among the 11 significant KEGG pathways observed for the predicted proteins with a high probability of interaction with A5 were found the PI3K-Akt signaling pathway, cAMP signaling pathway, and MAPK signaling pathway (Fig. 4H). The significant KEGG pathways associated with predicted proteins that interact with C1 or APO included chemical carcinogenesis, metabolism of xenobiotics by cytochrome P450, drug metabolism—cytochrome P450, cGMP-PKG signaling pathway and neuroactive ligand-receptor interactions (Fig. 4I and J). Likewise, the Gene Ontology (GO) Biological Process for the specific predicted proteins, based on PASS data, revealed that A5, C1 and APO had significantly common responses to chemical stimuli and cellular responses to dopamine (Suppl. Table S1).

Moreover, the SEA^30^ database predicted PDK2, NOTUM, DRD1 and MTNR1B as possible proteins interacting with A5 (Suppl. Figure S7A); while DRD1, DRD2, DRD3, DRD5, MTR1B, PARP1, PARP4, MMP15 and MMP26 were among the 30 proteins predicted to interact with C1 (Suppl. Figure S7B); and DRD1-5, PARP1 and PARP4 were among the 13 proteins predicted to interact with APO (Suppl. Figure S7C). Of importance, the SEA analysis showed that DRDs were among the proteins with a high probability of interaction with the compounds A5, C1 and APO (Suppl. Figure S7D). The protein–protein interaction network of the predicted proteins binding to A5, C1 and APO, revealed the groups of dopamine receptors and PARP1 in the network predicted for C1 and APO (Suppl. Figure S7E–G). However, considering that only four human proteins were predicted through SEA to associate with A5, no significant KEGG pathway was acquired for these proteins, although the pathways of neuroactive ligand-receptor interaction, dopaminergic synapse, and cAMP signaling were significantly related to the predicted proteins interacting with C1 and APO (Suppl. Figures S7H and I, respectively). Finally, as expected, the GO biological process showed that cellular response to dopamine was one of the most significant processes related to C1 and APO specific predicted proteins by SEA (Suppl. Table S2). Therefore, the use of the in silico prediction tools PASS and SEA reported not only proteins that are known to interact with APO, but likewise possible new proteins that may interact with APO, A5 and C1 (Fig. 4K). These data may expand the potential bioactivity of the aporphine and isoquinoline derivatives.

### ABCB1 expression correlates with TMZ resistance and A5 and C1 treatment lead to a reduction of ATP efflux from GBM cells

**Figure 5:**
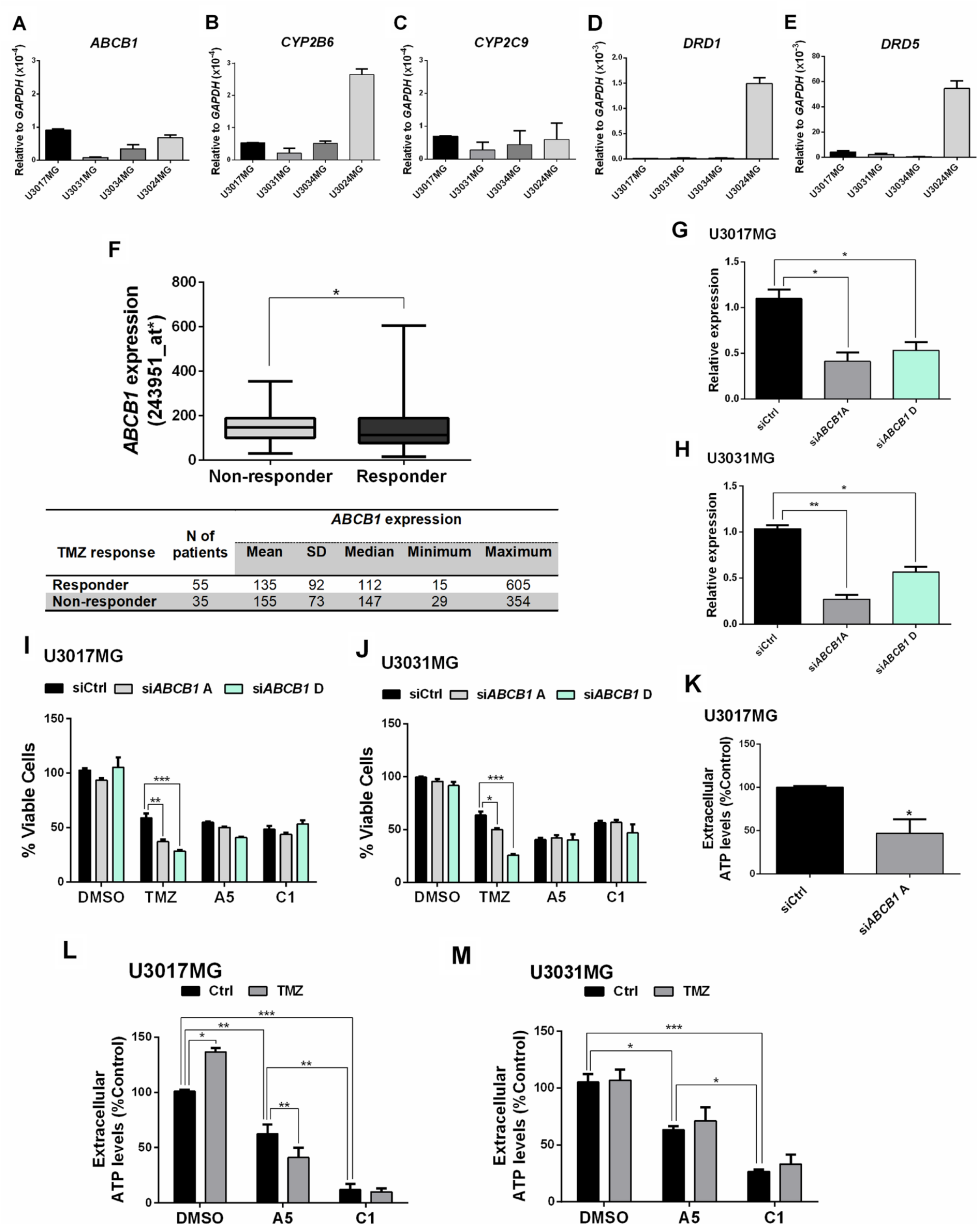
Characterization of predicted targets for aporphine and isoquinoline derivatives. RT-qPCR was used for detection of (**A**) *ABCB1*, (**B**) *CYP2B6*, (**C**) *CYP2C9*, (**D**) *DRD1* and (**E**) *DRD5* mRNA levels in U3017MG, U3031MG, U3034MG and U3024MG cells. (**F**) Correlation of TMZ response with *ABCB1* expression in tumor samples of GBM patients retrieved from http://www.rocplot.org/gbm. Asterisk on probe number indicates measurement by Affymetrix U133plus. GBM patients were categorized according to TMZ response and comparisons were performed with Mann–Whitney U-test (*p* = 0.0492). (**G**, **H**) RT-qPCR was used for detection of *ABCB1* after siRNA transfection in U3017MG (**G**) or U3031MG (**H**) cells. All the relative expression levels were normalized to *GAPDH* expression, and calculated using the 2^−ΔCt^ method. Error bars represent SD from three different experiments. (**I**, **J**) Viability level of U3017MG (**I**) or U3031MG (**J**) cells after treatment for 48 h with APO (25 µM), A5 (30 µM) or C1 (30 µM) of cells transfected with control (siCtrl) or *ABCB1*-specific siRNAs. Data were normalized against DMSO. (**K**—**M**) Extracellular ATP levels were measured in in U3017MG upon *ABCB1* silencing (**K**) and in U3017MG (**L**) and U3031MG (**M**) cells after treatment with A5 (30 µM) or C1 (30 µM) in the presence or absence of TMZ (150 µM) for 48 h and. Error bars represent SD from at least two biological replicates. Asterisks denote significance as determined by Welch t test, one-way ANOVA multiple comparisons followed by Bonferroni or Dunnet test (post-test) when appropriate; *p < 0.01, **p < 0.001, and ***p < 0.0001.

Five of the common predicted proteins interacting with A5 or C1 (namely ABCB1, CYP2B6, CYP2C9, DRD1 and DRD5) had their mRNA level assessed in the patient-derived GBM cells by RT-qPCR (Fig. 5A–E, respectively). Although the RT-qPCR analysis revealed a relatively low expression level for the five genes, we sought to correlate their expression with the TMZ response in GBM patients. Analysing the patient data available at Rocplot^31^, no significant correlation was observed among *DRD1*, *DRD5* and *CYP2B6* expression and patient response to TMZ (Suppl. Figure S8A–C, respectively). However, the higher expression of *CYP2C9* (median expression value = 22) was significantly correlated with response to TMZ therapy of GBM patients in comparison to those that did not respond (median expression value = 11) (Suppl. Figure S8D; *p* = 0.012). In addition, docking simulations of CYP2C9 with A5, (*R*;*S*)-C1, and (*R*;*S*)-APO were performed using 3D optimized structures of these compounds on the GOLD software. Favourable ΔG_bind_ energy for all evaluated compounds was observed with three conservative amino acid residues in common on the CYP2C9 catalytic site: Arg108, Phe114 and Leu208 (Suppl. Figure S9). This simulation suggested similar interactions between isomers in both cases with ΔG_bind_ energy values of -38.40 kcal/mol for (*S*)-C1, −36.62 kcal/mol for (*R*)-C1, −35.57 kcal/mol for (*R*)-APO and −35.45 kcal/mol for (*S*)-APO, while A5 had the highest ΔG_bind_ energy value (−26.76 kcal/mol), suggesting a lower affinity of CYP2C9 for A5 in comparison to (*R*;*S*)-C1 and (*R*;*S*)-APO (Suppl. Figure S9).

In contrast to the correlation between high *CYP2C9* expression and TMZ response, a significant correlation of *ABCB1* high expression was noted in patients not responding to TMZ (median expression value = 147) in comparison to the responder GBM patients (median expression value = 112) (Fig. 5F; *p* = 0.0492). Thus, to strengthen the relevance of ABCB1 for TMZ resistance on GBM cells, the silencing of *ABCB1* was performed using four different siRNAs (Suppl Figure S10A) and the knock-down was further validated on U3017MG and U3031MG transfected with siRNA (A and D) targeting the *ABCB1* transcripts (Fig. 5G and H, respectively). These GBM cells were subject to drug treatment with TMZ, A5 or C1, and the reduction of *ABCB1* expression significantly lead to a more potent TMZ but not to A5 or C1 response by U3017MG and U3031MG cells (Fig. 5I and J). Similar data was observed on U3034MG cells transfected with si*ABCB1* A (Suppl. Figure S10B and C). These data indicate that the cell death induced by A5 and C1 is not mechanistically dependent on the ABCB1 transporter, although our results pointed out that this protein was linked to TMZ resistance.

Nevertheless, since ATP efflux is one of the main functions of ABCB1, as noted by the significant decrease of extracellular ATP released by U3017MG upon knockdown of *ABCB1* (Fig. 5K), we sought to assess the levels of extracellular ATP in GBM cells before and after treatment with A5 or C1, combined or not with TMZ. Based on this, despite TMZ treatment inducing a significant increase in extracellular ATP levels in U3017MG but not in U3031MG, the GBM cells treated with A5 or C1, in the absence or presence of TMZ, showed a significant reduction of extracellular ATP amount in comparison to the control, with a stronger effect observed upon C1 treatment (Fig. 5L and M). Together, these results suggested that A5 and C1 might interact with ABCB1 blocking the efflux pump of ATP to the extracellular milieu, which consequently might enhance the TMZ effects on GBM cells.

**Figure 6:**
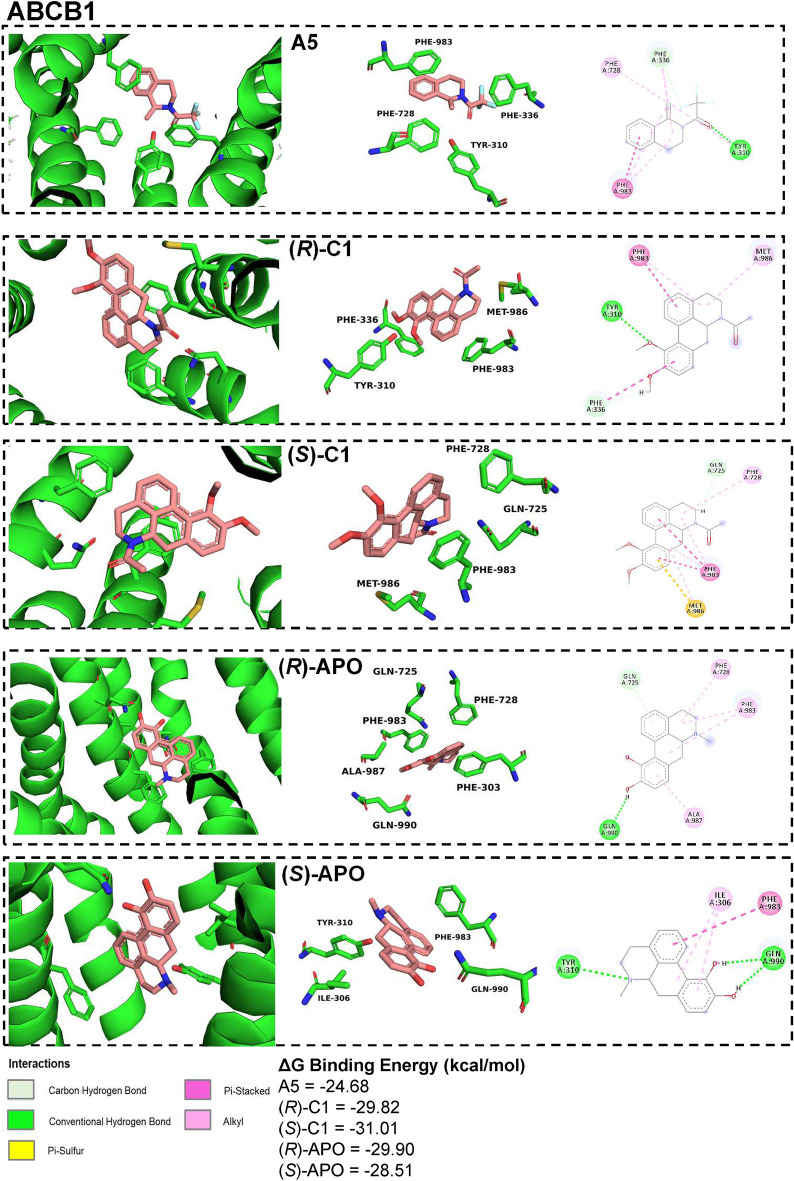
Docking simulations of ABCB1. Docking pose results in 3D and 2D representations of possible interactions of ABCB1 with A5, (*R*)-C1, (*S*)-C1, (*R*)-APO and (*S*)-APO. Images were created using Discovery Studio and the interactions with the different amino acids (A: position) are represented in different colors: gray (C-H bonds), green (H-bonds), lilac (Pi-Pi bonds), light purple (alkyl bonds) and yellow (pi-sulfur bonds).

Yet, docking simulations were performed to better understand the interaction of A5, (*R*;*S*)-C1, and (*R*;*S*)-APO with ABCB1. These data revealed favourable ΔG_bind_ values of the ABCB1 catalytic site to all three compounds evaluated but without dramatic differences among C1 and APO enantiomers: (*S*)-C1 −31.01 kcal/mol; (*R*)-C1 −29.82 kcal/mol; (*S*)-APO −28.51 kcal/mol; (*R*)-APO −29.90 kcal/mol (Fig. 6). The C1 enantiomers presented a higher affinity with the amino acids Phe983 and Met986 in the catalytic site of ABCB1, while APO enantiomers also had a higher affinity with Phe983, and an elevated hydrogen-bond affinity with Gln990. Nevertheless, A5 presented the lowest ΔG_bind_ value (− 25.44 kcal/mol), suggesting a lower affinity for ABCB1 in comparison to (*R*;*S*)-C1 and (*R*;*S*)-APO. Of note, Phe983 scored in all compound-protein interactions, suggesting that this is a possible key binding site residue that mediates a stable complex with the A5, C1 or APO (Fig. 6).

### TMZ co-treatment with aporphine and isoquinoline derivatives enhances cytotoxicity on GBM cells

**Figure 7:**
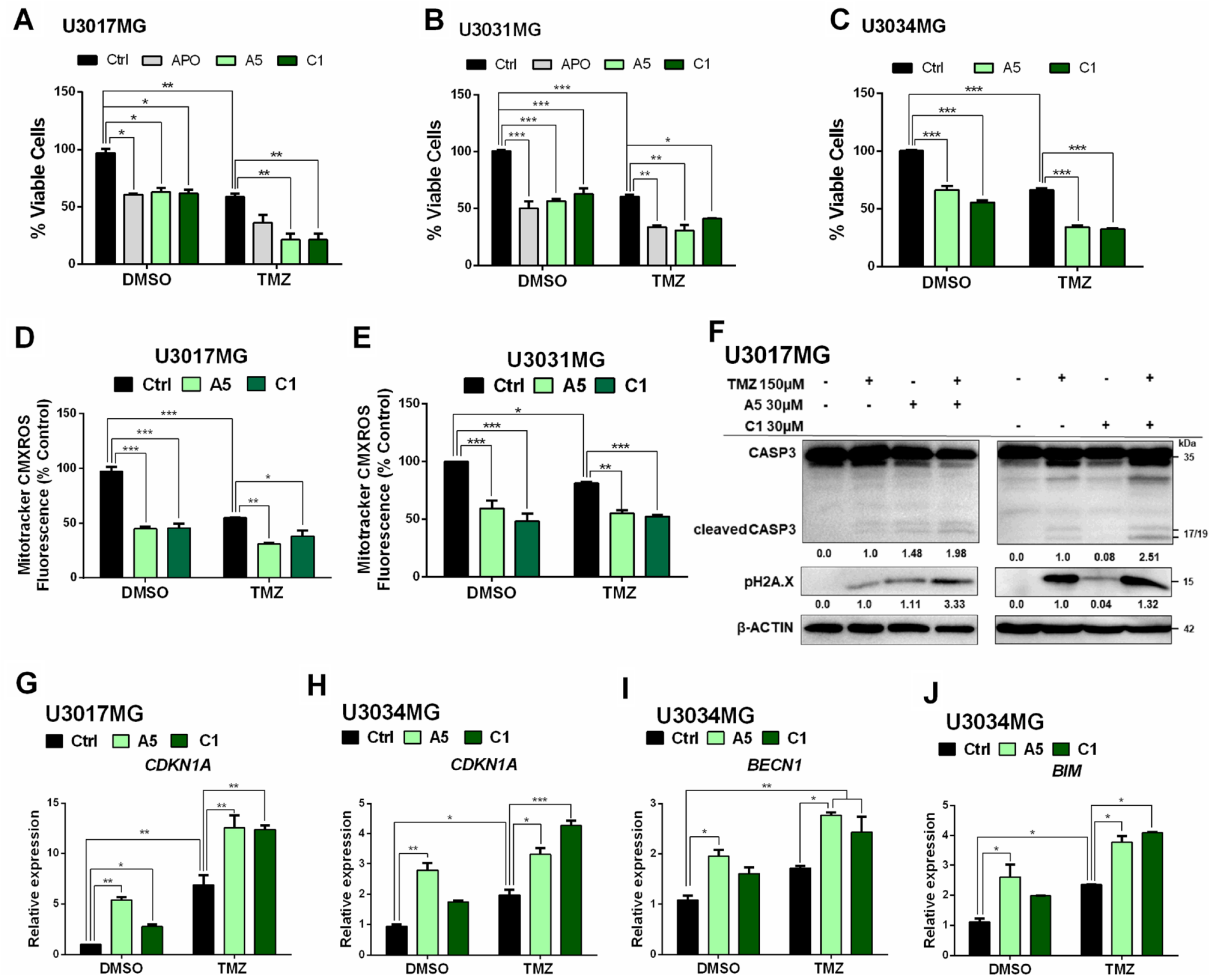
Aporphine and isoquinoline derivatives combined with TMZ increase cytotoxicity on GBM cells. Cell viability of U3017MG (**A**), U3031MG (**B**) and U3034MG (**C**) treated with APO (25 µM), A5 (30 µM) or C1 (30 µM) in the absence or presence of TMZ (150 µM) for 48 h was determined using the PrestoBlue reagent and normalized according to vehicle-treated control cells (DMSO). Mitochondrial transmembrane potential of (**D**) U3017MG and (**E**) U3031MG treated with A5 or C1 in the presence or absence of TMZ for 48 h was assessed by MitoTracker CMXROS and normalized against the vehicle-treated control cells (DMSO). (**F**) Immunoblot of cleaved caspase-3 and pH2A.X in U3017MG cells treated with A5 or C1 combined or not with TMZ for 48 h. β-Actin was used as loading control and molecular mass (kDa) markers are indicated along with densitometric values of normalized band intensity. The grouping of blots was cropped from different parts of the same gel. RT-qPCR was used for detection of (**G**, **H**) *CDKN1A* mRNA levels in (**G**) U3017MG and (**H**) U3034MG cells treated with A5 or C1 combined or not with TMZ for 48 h. (**I**, **J**) RT-qPCR was used for detection of (**I**) *BECN1* and (**J**) *BIM* in U3034MG cells treated with A5 or C1 combined or not with TMZ for 48 h. All the relative expression levels were normalized to *GAPDH* expression and calculated using the 2^−ΔΔCt^ method. Error bars represent SD from three biological replicates and *p* values were calculated according to the two-way ANOVA test followed by Bonferroni (post-test); *p < 0.05, **p < 0.01, ***p < 0.001.

New therapeutic approaches to increase TMZ toxicity on GBM cells are considered a matter of relevance towards translation from basic research to the clinic^32,33^. Hence, GBM patient-derived cells submitted to a 48-h regimen of TMZ in the presence of APO, A5 or C1 showed a significant decrease in the proportion of viable cells in comparison to exclusive TMZ treatment (Fig. 7A–C). Moreover, it has generally been assumed that dissipation of the mitochondrial potential is a relevant event in the apoptotic pathway^34^. Hence, the mitochondrial potential was measured in the patient-derived GBM cells treated with TMZ in the presence or absence of A5 or C1. In accordance with the cell viability data, TMZ therapy alone was able to decrease significantly the mitochondrial potential of GBM cells, as well as, treatments with A5 or C1. Furthermore, the co-treatment with TMZ and A5 or C1 showed an additional and significant mitochondrial potential reduction in comparison to the TMZ treatment alone (Fig. 7D and E), indicating a more potent induction of GBM cell death in the co-presence of these compounds.

Furthermore, to better characterize the effects of A5 and C1 treatment combined with TMZ, an evaluation of the expression of markers for cell death and DNA damage response was performed. This analysis pointed out higher levels of cleaved caspase-3 and pH2A.X in U3017MG cells treated with TMZ combined with A5 or C1 (Fig. 7F). In addition, *p21* mRNA expression was significantly increased by the co-treatment of TMZ with A5 or C1 in comparison to the respective controls (DMSO or TMZ alone) (Fig. 7G and H). The co-treatment also upregulated the expression of *BECN1* and *BIM* mRNA in U3034MG cells (Fig. 7I and J). It is therefore evident that combinatorial treatment of GBM cells with TMZ and A5 or C1 provides promising indication for future in vivo testing.

## Discussion

To identify new drugs for GBM treatment, we tested 12 compounds obtained through APO total synthesis^25^. Apart from validating the cytotoxic effect of APO on patient-derived GBM cells, as previously reported by others^21,22^, our data revealed that A5 and C1 are two new promising compounds presenting effective toxic activity against GBM cells. To date, the cytotoxic mechanisms induced by aporphine alkaloids have not been fully described. Notwithstanding, the proposed cytotoxicity induced by APO was associated with its autoxidation, driving ROS formation^23^. The presence of ROS might lead to a downregulation of genes linked to mitochondrial energy metabolism^20^, such as ATP synthases *ATP5O* and *ATP5D*, cytochrome oxidases *COX8A* and *COX7* and NADH dehydrogenases *NADUFS8* and *NADUFB2* or via ROS intercalation into the DNA double helix promoting tumor cell death^35^. Besides, it was reported that apomorphine can bind to the Cys77 of the ubiquitin ligase MDM2, leading to an inhibitory effect against MDM2-p53 interaction, which might avoid p53 degradation by MDM2, contributing to the growth-suppressive function of p53^36^. The function of apomorphine promoting p53 activity has a promising clinical relevance, since p53 inactivation by MDM2 is a frequent event in primary GBMs^37^. Notably, in the context of HNSCC, we found APO, C1 (1-(10,11-dimethoxy-6a,7-dihydro-4*H*-dibenzo[*de*,*g*]quinolin-6(5*H*)-yl)ethanone) and A5 (2,2,2-trifluoro-1-(1-methylene-3,4-dihydroisoquinolin-2(1*H*)-yl)ethanone) exerting cytotoxic activity through DNA damage and apoptotic induction through caspase-3 pathway activation^24^.

A major route leading to caspase activation is initiated by the cytochrome c pathway^38^ and GBM cells subjected to A5 or C1 treatment presented COX activity significantly increased, indicating a higher release of intrinsic pro-apoptotic factors from their mitochondria. In line with this data, the induction of caspase-3 cleavage and the enhanced expression of apoptotic-related genes, such as *BIM* and *BECN1* was evident after GBM cells were treated with A5, C1 or APO. It is known that Beclin 1 has a central role in autophagy and does not function as a pro-apoptotic molecule, even when overexpressed^39^. Nevertheless, the activation of caspase3 after A5, C1 or APO treatment can mediate Beclin 1 cleavage, leading to the translocation of beclin 1 C-terminal fragments to the mitochondria, which promotes the crosstalk between apoptosis and autophagy pathways^39^. Although we noted *BAX* mRNA expression increased particularly after A5 treatment, it is important to mention that apoptosis induced by Bax protein can decrease autophagy by increasing caspase-mediated cleavage of Beclin^40^. Moreover, DDR activation can lead to the signaling of cell death mediated by apoptosis^41,42^. Hence, our results also pointed out to DDR machinery activation on GBM cells treated with A5 or C1, as evidenced by the increase of *p21* levels, which suggests activation of p53 signaling followed by repression of *CCNB1* expression. Collectively, the Fig. 2 shows that the cell death induced by A5 or C1 represents an amplified loop to induce apoptosis in GBM cells.

Different signaling pathways (e.g. platelet‐derived growth factor, transforming growth factor‐β, leukemia inhibitory factor, Wnt, epidermal growth factor and Notch) can drive the formation of neural/glial stem cells during GBM development, contributing to a diverse variety of stem‐like cells with the capacity of self-renewal, proliferation and differentiation^43–45^. Although TMZ transiently suppressed proliferative GBM cells, a subpopulation of TMZ-resistant cells rises, exhibiting GSC properties, sustaining long-term tumor growth and leading to GBM progression after TMZ administration^4^. Based on this, the assessment of the effect of aporphine and isoquinoline derivatives on the formation of GSCs revealed that the cytotoxicity induced by these compounds effectively blocked the formation of gliomaspheres. Consistent with this, a reduction in the protein and transcript levels of GBM stemness markers (NESTIN, CD133 and SOX2) was observed after treatment with these nitrogenous heterocyclic compounds. It is worth mentioning that a correlation between high levels of GBM stemness gene expression and poor survival of GBM patients has already been reported^46^. Moreover, the strongest effect of these compounds downregulating NESTIN protein expression rather than in their transcripts suggest a post-transcriptional regulation, but not evaluated here. In addition, the astrocytic protein GFAP, which is a standard diagnostic marker for GBM^46^ and reported previously expressed in neuronal stem cells from postnatal and adult brain^47^, was shown downregulated after treatment with the aporphine and isoquinoline derivatives but not by TMZ, indicating different signaling effects of A5 and C1 in comparison to TMZ on GSCs. It cannot go unnoticed that the effect of these compounds on GBM cells was not evaluated on established gliomaspheres or after a period longer than one week. Altogether, the results in Fig. 3 indicate the ability of A5, C1 and APO to impair gliomasphere growth, which might lead to a lower chemo-resistant GBM phenotype.

In silico tools (i.e. PASS and SEA) were applied to predict the pharmacological activities and the biologically active substrates for A5, C1 and APO, based on their structure. The data retrieved from the PASS database for APO pharmacological activities predicted the established effect of APO as a dopamine antagonist and tyrosine hydroxylase inhibitor^14,15,48^, besides acting as an antineoplastic alkaloid^17–24^. Likewise, dopamine antagonism, tyrosine hydroxylase inhibition and antineoplastic alkaloid activity were predicted as pharmacological activities for C1 as well, considering its similar structure to APO. In contrast, apart from the neurotransmitter antagonistic effect that was found commonly for the three compounds, an antineoplastic enhancer activity was predicted for A5. Of note, the anti-cancer activity predicted for A5, C1 and APO agreed with our in vitro data; thus, identifying the predicted proteins interacting with these compounds can guide us to understand the mechanism by which these compounds may exert anti-cancer properties in tumor cells. Although this prediction was based on the 2D structure of these compounds, several proteins were likely interacting with A5 (PASS = 164 and SEA = 4), C1 (PASS = 70 and SEA = 30) and APO (PASS = 146 and SEA = 13). Interestingly, a fraction of these predicted proteins was associated with base excision repair, chemical carcinogenesis, metabolism of xenobiotics by cytochrome P450 and neuroactive ligand-receptor interaction.

In the context of neurotransmitter-mediated regulation of cancer phenotypes, dopamine receptors DRD1 and DRD5 (D1-like subfamily: coupled to Gα_s_ or Gα_lf_ proteins), whose transcripts were expressed in the GBM patient-derived cells evaluated here, and also DRD2 and DRD3 (D2-like subfamily: coupled to Gα_i/o_) were among the possible proteins interacting with A5 (D1-like subfamily only), C1 or APO. It is important to mention that the role of the D1-like receptors in GBM tumorigenesis is still contradictory^49^. Although *DRD1* and *DRD5* expression does not appear to be associated with TMZ response in GBM patients, gene expression data retrieved from The Cancer Genome Atlas (TCGA) correlated high *DRD1* and *DRD5* expression with worse overall survival of GBM patients^50^, while DRD5 activation inhibited GBM growth by inducing autophagic cell death^51^. Notwithstanding, the high level of *DRD2* expression was correlated with the worst survival of GBM patients^48^ and the use of DRD2 antagonists (e.g. haloperidol^52^ and trifluoperazine^22,53^) suppressed the growth of GBM cells by inhibiting mitogenic signaling, and impaired gliomasphere formation, followed by reduction of SOX2 levels, which improved the survival of GBM-mouse models treated with radiotherapy^53^. Although the data presented here is in line with the use of DRD2 antagonists suppressing GBM tumorigenesis, further studies are still required to characterize the interaction of dopamine receptors with A5 and C1.

Another promising pharmacological effect predicted for the aporphine and isoquinoline derivatives studied here was related to the metabolism of xenobiotics by the cytochrome P450 system, with CYP2B6 and CYP2C9 as common targets for these compounds. Noteworthy, both CYP2B6, which is the only functional enzyme from the CYP2B subfamily, and CYP2C9, which is the most abundant CYP2C isoform in the liver, play a relevant role in the oxidation of anticancer agents, such as cyclophosphamide and ifosfamide^54^. For instance, CYP2B6 overexpression in primary murine gene-engineered neural stem/progenitor cells, used as a therapeutic delivery system to an in vivo GBM model, increased the activation of cyclophosphamide, resulting in higher rates of death^55^. Moreover, CYP2C9 catabolizes the conversion of aspirin to gentisic acid, which blocks the binding of fibroblast growth factor to its receptor, thus repressing growth of C6 glioma cells^54^. Nevertheless, although the expression of *CYP2B6* and *CYP2C9* were evaluated in the GBM patient-derived cells, only the higher expression of *CYP2C9* was correlated with the TMZ response of GBM patients. Since the prediction tools did not calculate the molecular energy levels, docking simulations were performed to better characterize the predicted interactions of CYP2C9 with A5, (*R*;*S*)-C1 and (*R*;*S*)-APO. These analyses revealed favourable ΔG_bind_ energy for these compounds, sharing among them three conservative amino acids on the catalytic site of CYP2C9 (Arg108, Phe114 and Leu208); however, whether such interactions are inducing or blocking the activity of this enzyme still remains to be addressed.

The ATP-dependent translocase ABCB1, also known as multidrug resistance protein 1 (MDR1) was another common predicted target for A5, C1 and APO. ABCB1 is a major ATP-binding cassette transporter of the brain-blood barrier and regulates the trafficking of multiple drugs out of brain tumor cells, including TMZ^56,57^. Although *ABCB1* mRNA was poorly expressed in the patient-derived GBM cells studied here, by using available cancer patient databases^31^, we were able to correlate significantly the high expression of *ABCB1* with TMZ resistance in GBM patients. Additionally, upon *ABCB1* silencing, GBM cells presented a higher TMZ cytotoxic effect possibly due to an increase of TMZ intracellular concentration, as reported by others through genetic perturbations or by blocking ABCB1 activity with chemical inhibitors (e.g. PSC833, reversan, CP-100356, and elacridar)^58–60^. Nevertheless, although ABCB1 levels did not influence the response of GBM cells to A5 or C1, the fact that these compounds were predicted to interact with ABCB1, which increases TMZ resistance in GBM cells, lead us to investigate better formation of such complexes through molecular modelling simulations. Thus, predicted favourable ΔG binding energy supports possible interaction of ABCB1 with A5, (*R*;*S*)-C1 and (*R*;*S*)-APO, and identified Phe983 as a specific amino acid present in all compound-protein interfaces. Additionally, GBM cells treated with A5 or C1, combined or not with TMZ, reduced significantly the levels of extracellular ATP, suggesting the blockage of ABCB1 activity in GBM cells by the treatment with these compounds. The lower ΔG binding energy required for C1 interaction with ABCB1 underscores the stronger interaction with this protein, followed by an impressive reduction of ATP release in comparison to the cells treated with A5. Notably, TMZ treatment alone increased ATP released from U3017MG cells, which might indicate stronger ABCB1 activity contributing to a reduction in the intracellular concentration of TMZ.

A plethora of signaling pathways driving DNA repair, formation of GSC subpopulations, and self-defence mechanisms, including the ATP-binding cassette transporters, are some of the mechanisms leading to TMZ resistance in GBM cells^4,33,34,58–60^. Hence, by overcoming such resistance mechanisms, the relevance of our findings was underscored as shown by the significant increment of cytotoxicity and cell death induced on GBM cells that were treated with A5 or C1 in combination with TMZ. The additive toxic effect was observed through the reduction of cell viability and mitochondrial potential, which was correlated with the increment of cleaved caspase-3, and *BECN1* and *BIM* expression, when compared to TMZ treatment alone, indicating that cell death was mediated through apoptosis. Moreover, the induction of DDR, as noted by enhanced levels of pH2A.X and *p21*, suggested more DNA damage once the cells were treated with A5 or C1 combined with TMZ. To date, because the regimens of salvage chemotherapies in GBM patients have a limited impact on overall survival, new therapeutical approaches that do not have cross-resistance with alkylating agents and increase TMZ toxicity on GBM cells are urgently needed^32,33^.

The compounds A5 and C1 were intermediates in the convergent total synthesis of (±)-apomorphine hydrochloride, which was achieved after nine steps in an overall yield of 8%^25^. Of importance, compound A5 was acquired after three steps in 84% yield, while C1 was obtained after six steps in 39% yield. Hence, APO synthesis is much less efficient in comparison to its intermediates A5 and C1 and the large-scale production of A5 and C1 is considered promising mainly due to the pharmacological potential demonstrated against GBM cells, by increasing cell death and impairing GSC formation. The pharmacological activities driven by these compounds are still not well understood but antagonism of dopamine receptors, activation of the cytochrome P450 system and blockage of ABCB1 activity may be plausible mechanisms induced by A5 and C1 treatments, which also lead to an additive toxic effect on patient-derived GBM cells co-treated with TMZ. Therefore, although further in vivo studies are still required to confirm the effects of A5, C1 and APO on GBM and non-tumorigenic cells, our data support a potential therapeutic application of A5 and C1 as new cytotoxic agents against GBM tumors.

## Materials and methods

### Cell culture and viability

The GBM and non-tumorigenic cell lines used in this study were either obtained from ATCC (A172; CRL-1620, T98G; CRL-1690 and HEK-293 T; CRL-3216) or were a generous gift from the human GBM cell culture resource (Human glioblastoma cell culture—HGCC: https://www.hgcc.se/), ^26^ which collects and authenticates primary cells isolated from grade IV glioma biopsies (patient-derived U3017MG/Classical, U3024MG/Mesenchymal, U3031MG/Mesenchymal, and U3034MG/Mesenchymal). Ethical protocols and permission have been secured by the biobank approved by the Uppsala Län regional ethical review board after written consent by all patients, as described^26^. Of note, all methods were carried out in accordance with relevant guidelines and regulations. The commercial cell lines were cultured in DMEM (ThermoFisher Scientific), with 10% fetal bovine serum (ThermoFisher Scientific) and 0.01 µg/ml of penicillin–streptomycin (Sigma-Aldrich). Patient-derived cells were cultured on laminin (Sigma-Aldrich), in a 1:1 ratio of DMEM/F12 Glutamax with Neurobasal medium (ThermoFisher Scientific) supplemented with B27 and N2 (both from ThermoFisher Scientific), penicillin/streptomycin, 10 ng/ml EGF and fibroblast growth factor 2 (FGF2) (PeproTech EC Ltd). GBM cells were grown in an incubator at 37 °C with 5% CO_2_.

The drug toxicity was assessed through cell viability after treatment and determined by PrestoBlue Cell Viability Reagent (ThermoFisher Scientific), following the manufacturer's instructions, as previously described^24,45^. The proportion of viable cells observed in the treatment with 0.1% DMSO alone (drug vehicle) was considered 100% and the relative fluorescence units from treated cells were normalized according to vehicle-treated control cells. The 50% inhibitory concentration (IC_50_) for the compounds was estimated from dose–response curves. Experiments were performed in triplicate and data are expressed as mean ± SD.

### Chemical substances

2-phenethylamine (**A1**), *N*-phenethylacetamide (**A2**), 1-methyl-3,4-dihydroisoquinoline (**A3**), 1-(1-methylene-3,4-dihydroisoquinolin-2(1*H*)-yl)ethanone (**A4**), 2,2,2-trifluoro-1-(1-methylene-3,4-dihydroisoquinolin-2(1*H*)-yl)ethanone (**A5**), 3,4-dimethoxybenzaldehyde (**B1**), 3,4-dimethoxyphenol (**B2**), (3,4-dimethoxy-2-(trimethylsilyl)phenoxy)trimethylsilane (**B3**), 3,4-dimthoxy-2-(trimethylsilyl)phenol (**B4**), 1-(10,11-dimethoxy-6a,7-dihydro-4*H*-dibenzo[*de*,*g*]quinolin-6(5*H*)-yl)ethanone (**C1**), 10,11-dimethoxy-5,6,6a,7-tetrahydro-4*H*-dibenzo[*de*,*g*]quinolone (**C2**), and 10,11-dimethoxy-6-methyl-5,6,6a,7-tetrahydro-4*H*-dibenzo[*de*,*g*]quinoline (**C3**) were obtained through the convergent total syntheses of APO^25^ and the structures of these compounds were detailed in Suppl. Figure S1A^24^. The in silico prediction tool (http://www.cbligand.org/BBB) ^61^ was used to examine the possibility of these compounds to pass through the brain-blood barrier (BBB). Dimethyl sulfoxide (DMSO) was used to prepare the drug stocks (100 mM) and all the working dilutions were set in culture medium. Temozolomide (TMZ) and lomustine were purchased from SantaCruz Biotechnology (#sc-203292 and #sc-202697, respectively).

### RNA extraction, cDNA synthesis and RT-qPCR

RNA extraction and real time reverse‐transcription polymerase chain reaction (RT-qPCR) were performed as previously described^45^. Expression levels of the genes evaluated in this study was determined by RT-qPCR using *glyceraldehyde-3-phosphate dehydrogenase* (*GAPDH*) levels to normalize the expression. The primer sequences of the genes are described in Suppl. Table S3.

### Immunoblotting

Immunoblotting were performed as previously described^45^. The protein content was determined using Bradford Reagent (#5000006; Bio‐Rad) and the antibodies used were purchased from Cell Signaling: anti-caspase3 (#9662) and anti-pH2A.X (#9718); Millipore: anti-nestin (#MAB5326), anti-CD133 (#MAB4399), and anti-SOX2 (#AB5603); or from Santa Cruz Biotechnology (Dallas, USA): anti-GFAP (#sc-6171-R). ImageJ bundled with Java 1.8.0_172 (U. S. National Institutes of Health, Maryland, USA) was used to normalize intensity levels according to loading controls expression blotted with anti‐GAPDH (#AM4300; ThermoFisher Scientific) or anti‐β‐actin (#sc-69879; Santa Cruz Biotechnology). Original immunoblots are represented in Suppl. Figure S11.

### Cytochrome C oxidase activity assay

The Cytochrome C Oxidase Assay Kit (#ab239711; Abcam) was used to identify the release of pro-apoptotic factors by the mitochondria of GBM cells. Briefly, 5 × 10^6^ cells were incubated with DMSO, A5 (30 µM) or C1 (30 µM). After 24 h treatment, the cells were collected and mitochondrial extraction was processed with Mitochondria Isolation Kit (#89874; ThermoFisher Scientific). After protein quantifications, total purified mitochondrial was used to measure COX activity. Then, to characterize the enzyme activity over a 30 min period, the oxidation of reduced cytochrome c with an absorbance decrease at 550 nm was measured. The rate of the enzyme reaction was calculated in the linear range (n = 2 for each treatment condition) and normalized per protein amount, according to the manufacturer.

### Sphere and extreme limiting dilution analysis (ELDA)

Single‐cell suspensions were seeded with decreasing numbers, 64‐1 cells/well, in low‐attachment 96‐well plates (#3474; Corning Inc.) in 200 μl GBM cell media with or without TMZ (125 µM), A5 (30 µM), C1 (30 µM) or APO (25 µM). Each dilution included 6 replicates per experiment. On Day 7, spheres larger than 50 μm in diameter were considered and wells containing spheres were scored for each condition to estimate the stem cell frequency and analyzed by the ELDA software (https://bioinf.wehi.edu.au/software/elda/) ^28^.The results are plotted as log‐fraction of wells without spheres as a function of the plated cell number, as previously described^44^.

### Computational target prediction

To predict mechanistically the biological activity of various compounds, two online tools were used for target fishing: PASS (http://way2drug.com/PassOnline) ^29^ and SEA (https://sea.bkslab.org) ^30^. PASS, apart from predicting direct protein interaction, also predicts the spectrum of pharmacological activity and toxic effect as probable activity (Pa) and probable inactivity (Pi) with accuracy of prediction as high as 85%. The ratio of Pa and Pi for a compound was considered to describe such compounds. Direct protein interaction predicted through PASS was considered with confidence > 0.2. No cut-off value was applied for proteins predicted through SEA, considering the lower amount of proteins predicted. The potential directly-interacting proteins acquired with the different software was analyzed. Moreover, a protein–protein interaction network for the specific proteins interacting with each compound was constructed using the Search Tool for the Retrieval of Interacting Genes database (STRING—version 10.5; http://string-db.org/), with the required high confidence score (> 0.7). Subsequent KEGG pathway and GO-biological process enrichment analyses were performed.

### Correlation analysis of gene expression

Correlation of gene expression and TMZ response in GBM patients was conducted using the database from ROC Plotter (http://www.rocplot.org/gbm) ^31^. GBM patients were categorized as responders or non-responders, according to the survival status at 16 months’ post-surgery.

### Molecular docking study

The docking simulations of A5, (*R*;*S*)-C1, and (*R*;*S*)-APO with ABCB1 and CYP2C9 were performed using the software GOLD v. 2021.3.0. The molecular structures were drawn using ChemDraw and converted in a 3D mol2 file with the PyMOL software. The spatial interactions of C1 and APO enantiomers were considered, since their arrangement is a determining factor for each ligand activity, and consequently can lead to ΔG_bind_ energy variations. The 3D protein structure of these proteins was retrieved from RCSB Protein Data Bank (https://www.rcsb.org/) (PDB code: 6QEX and 3IBD, respectively). The docking protocols were validated by redocking for each protein (Suppl. Figure S12). The grid box determined was placed at each ligand binding site. The molecular docking of ABCB1 was performed with the center of the grid box at x = 172.811, y = 166.377 and z = 160.048 with an approximated radius of the binding site of 6 Å with a total of 20 runs. For CYP2C9, the center of the grid box was set at x = −35.815, y = 61.358, and z = 5.716 with radius of 8 Å and 20 runs. Visualizations were created with Discovery Studio 2019 as well as PyMOL and the data were analyzed based on the best-ranked pose of each case measuring interactions bonds and calculated ΔG_bind_ energy.

### siRNA transfection analysis

Once reached 70% of confluence, GBM cells were transfected with siLentFect (#170-3360; Bio‐Rad Laboratories AB) and the respective siRNA (20 nM), according to the manufacturer. After 48 h, the cells were collected for knockdown validation and seeded for further experiments. The human‐specific siRNA oligonucleotides (QIAGEN) were: a non-mammalian siRNA (#SI03650325) and a set of four different siRNA against the target mRNA (si*ABCB1* A: #SI00018718–ATCGAGTCACTGCCTAATAAA; si*ABCB1* B: #SI00018732–GACAGAAAGCTTAGTACCAAA; si*ABCB1* C: #SI03028116–AACATTCGCTATGGCCGTGAA; si*ABCB1* D: #SI03040156–ACCGGACATCCCAGTGCTTCA).

### Extracellular ATP analysis

Forty-eight hours after treatment with the compounds in the absence or presence of TMZ, the conditioned media were collected, centrifuged for 5 min at 300 × *g* and boiled for 5 min at 95 °C for extracellular ATP analysis with the ATP Determination Kit (#A22066; ThermoFisher Scientific), following the manufacturer’s instructions. Data are expressed as percentage relative to the control cells after correction for protein content from the respective cells that generated the conditioned media.

### Mitochondrial transmembrane potential analysis

Mitochondrial membrane potential was analyzed fluorimetrically with MitoTracker™ Red CMXRos (#M7512; ThermoFisher Scientific), as previously described^45^. Briefly, after 48 h-treatment with the compounds in the absence or presence of TMZ, GBM cells were incubated with 500 nM final probe concentration in Hank’s balanced salt solution without phenol red for 30 min at 37 °C, detached with accutase, washed with PBS, and resuspended in the same media, transferred in duplicate into a 96‐well plate, and recorded the fluorescence (Ex/Em 579/599 nm). Data are expressed as a percentage relative to the control after correction for protein content.

### Statistical analyses

All data are presented as the mean ± standard deviation. The statistical analysis obtained after the cells were subjected to cytotoxic treatment was performed using Welch t test, besides one-way or two-way ANOVA, followed by multiple paired comparisons conducted by means of the Dunnet or Bonferroni's post-test method when applicable. Mann–Whitney test was used to evaluate the associations between gene expression and TMZ treatment response. A *p* value < 0.05 was necessary to determine all statistically significant differences.

### Ethics approval

All experimental protocols were approved by the institutional ethics committee.

## Supplementary Information

Supplementary Legends.

Supplementary Information.

## Data Availability

The datasets used and/or analysed during the current study available from the corresponding author on reasonable request.
