# Supplementary material for: Aporphine and isoquinoline derivatives block glioblastoma cell stemness and enhance temozolomide cytotoxicity
Source: Sci Rep. 2022 Dec 7;12:21113. doi: 10.1038/s41598-022-25534-2 (PMC9729571; doi:10.1038/s41598-022-25534-2)
Supplement: Supplementary file 1 — Supplementary Legends. [file 41598_2022_25534_MOESM1_ESM.docx]

**Supplementary table legends**

**Suppl. Table 1.** List of significant Top 15 Gene Ontology (GO) Biological Processes identified through the STRING database (Protein Analysis Through Evolutionary Relationships) with the predicted proteins which directly interact with A5, C1 or APO compounds, according to the Prediction of Activity Spectra for Substances (PASS) software (confidence > 0.2).

**Suppl. Table 2.** List of significant Top 15 Gene Ontology (GO) Biological Processes identified through the STRING database (Protein Analysis Through Evolutionary Relationships) with the predicted proteins which directly interact with C1 or APO compounds, according to the Similarity ensemble approach (SEA) software.

**Suppl. Table 3.** Primer sequences used for RT-qPCR assays.

**Supplementary figure legends**

**Suppl. Figure 1.** (**A**) Chemical structures of the substances assessed in this study. (**B**) *In silico* prediction scores of A5, C1 and APO for transport across the Brain Blood Barrier (BBB), according to http://www.cbligand.org/BBB^60^.

**Suppl. Figure 2.** Effect of TMZ on GBM cells viability. Antiproliferative effect of TMZ on GBM cells was determined by the PrestoBlue cell viability test in 48-h culture assays. The error bars represent the standard deviation of three independent assays

**Suppl. Figure 3.** *CCNB1* expression in U3017MG cells. RT-qPCR was used for detection of *CCNB1* mRNA levels in U3017MG cells treated with DMSO, TMZ (150 µM), A5 (30 µM), C1 (30 µM), or APO (25 µM) for 48 h. The relative expression levels were normalized to *GAPDH* expression and calculated using the 2^−ΔΔCt^ method. Error bars represent the standard deviation from three biological replicates (*p < 0.05, **p < 0.01).

**Suppl. Figure 4.** *In silico* predictions of toxic effects for the use of aporphine and isoquinoline derivatives. Prediction of Activity Spectra for Substances (PASS; http://way2drug.com/PassOnline) identified the toxic activity of tested compounds (**A**) A5, (**B**) C1 and (**C**) APO. The data were represented as the ratio of probable activity (Pa) per probable inactivity (Pi) for each pharmacological activity.

**Suppl. Figure 5.** Distribution of the predicted proteins through PASS interacting with A5, C1 and APO. Only the proteins with confidence > 0.2 were considered for further analysis.

**Suppl. Figure 6.** Protein-protein interaction (PPI) network construction by STRING using the PASS predicted proteins interacting with (**A**) A5, (**B**) C1 and (**C**) APO.

**Suppl. Figure 7.** *In silico* predictions of the target proteins for the aporphine and isoquinoline derivatives. The similarity ensemble approach (SEA; https://sea.bkslab.org/) identified the predicted proteins interacting with (**A**) A5, (**B**) C1 and (**C**) APO. (**D**) Distribution of the 39 predicted proteins through SEA interacting with A5, C1 or APO. The table lists the common proteins for A5, C1 and APO; A5 and APO; C1 and APO. Protein-protein interaction (PPI) network construction by STRING to the SEA predicted proteins interacting with (**E**) A5, (**F**) C1 and (**G**) APO. Representation of the significant KEGG pathways identified through the STRING database related to the SEA predicted proteins interacting with (**H**) C1 and (**I**) APO.

**Suppl. Figure 8.** Gene expression correlation with response of GBM patients to TMZ therapy. Comparison between expression of (**A**) DRD1, (**B**) DRD5, (**C**) CYP2B6 and (**D**) CYP2C9 in tumor samples of GBM patients presenting response to TMZ and no response to TMZ. The number of patient samples included in the analysis along with the p-value indicated, according to Mann-Whitney U-test.

**Suppl. Figure 9.** Docking simulations of CYP2C9. Docking pose results in 3D and 2D representations of possible interactions of CYP2C9 with A5, (*R*)-C1, (*S*)-C1, (*R*)-APO and (*S*)-APO. Images were created using Discovery Studio and the interactions with the different amino acids (A: position) are represented in different colors: blue (Halogen), green (H-bonds), gray (C-H bonds), lilac (Pi-Pi bonds), and light purple (alkyl bonds).

**Suppl. Figure 10.** (**A**, **B**) RT-qPCR was used for detection of *ABCB1* after siRNA transfection in U3017MG (**A**) or U3034MG (**B**) cells. All the relative expression levels were normalized to *GAPDH* expression, and calculated using the 2^−ΔΔCt^ method. Error bars represent SD from three different experiments. The relative expression levels were normalized to *GAPDH* expression and calculated using the 2^−ΔΔCt^ method. (**C**) Viability level of U3034MG cells transfected with control (siCtrl) or *ABCB1*-A after treatment for 48 h with TMZ (150 µM), A5 (30 µM) or C1 (30 µM). Data were normalized against DMSO. Error bars represent SD from at least two biological replicates. Asterisks denote significance as determined by Welch t test or one-way ANOVA multiple comparisons followed by Dunnet test (post-test) when appropriate (*p < 0.01, **p < 0.001).

**Suppl. Figure 11.** Unprocessed immunoblots. The unprocessed immunoblots corresponding to the three indicated figures are shown. Rectangles indicate the cropped part of the immunoblot used in each of the main figure. The figure layout, densitometric values and molecular size markers are indicated exactly as in the main figures. N.S.: non-specific protein band detected by the antibodies used.

**Suppl. Figure 12.** Redocking simulations of ABCB1 and CYP2C9. (**A**) Redock structure of ABCB1 crystal interacting with taxol with root-mean-square deviation (RMSD) = 6.62. (**B**) Redock structure of CYP2C9 crystal interacting with ethyl {2-[([1,3]thiazolo[4,5-c]pyridine-2-carbonyl)amino]thiophene-3-carbonyl}carbamate with RMSD = 6.30.
