# Supplementary material for: Aporphine and isoquinoline derivatives block glioblastoma cell stemness and enhance temozolomide cytotoxicity
Source: Sci Rep. 2022 Dec 7;12:21113. doi: 10.1038/s41598-022-25534-2 (PMC9729571; doi:10.1038/s41598-022-25534-2)
Supplement: Supplementary file 2 — Supplementary Information. [file 41598_2022_25534_MOESM2_ESM.pdf]

Suppl. Table 1

Term Description	FDR q-value	Matching Proteins
**A5**		
Adenylate cyclase-inhibiting g protein-coupled acetylcholine receptor signaling pathway	4.28E-08	CHRM3,CHRM1,CHRM5,CHRM2,CHRM4
Acetylcholine receptor signaling pathway	4.28E-08	CHRM3,CHRM1,CHRM5,CHRM2,CHRNA7,CHRM4
Cellular response to oxygen-containing compound	4.28E-08	PPP5C,APEX1,CHRM3,MAPK3,PRKCI,CHRM1,CDK19,HRH2,CHRM5,CHRM2,ESR1,CHRNA7,CHRM4,NR4A1
Cellular response to dopamine	4.28E-08	CHRM3,MAPK3,CHRM1,HRH2,CHRM5,CHRM2,CHRM4
Cellular response to stimulus	6.59E-08	PPP5C,APEX1,PARP2,CHRM3,CYP2C9,MAPK6,MAPK3,CAMK4,PRKCI,CHRM1,OXSR1,CYP2B6,CDK19,ZAK,HRH2,CHRM5,TSSK1B,CIT,MAP2K7,CHRM2,ESR1,CHRNA7,CHRM4,NR4A1
Cellular response to nitrogen compound	1.54E-07	APEX1,CHRM3,MAPK3,PRKCI,CHRM1,HRH2,CHRM5,CHRM2,CHRNA7,CHRM4,NR4A1
Response to stimulus	2.12E-07	PPP5C,APEX1,PARP2,CHRM3,CYP2C9,MAPK6,MAPK3,CAMK4,PRKCI,CHRM1,OXSR1,CYP2B6,CDK19,ZAK,HRH2,CHRM5,TSSK1B,CIT,MAP2K7,CHRM2,ESR1,CHRNA7,CHRM4,NR4A1,ABCB1
Cellular response to organic cyclic compound	5.21E-07	APEX1,CHRM3,MAPK3,CHRM1,HRH2,CHRM5,CHRM2,ESR1,CHRM4,NR4A1
Response to organonitrogen compound	6.78E-07	PPP5C,APEX1,CHRM3,MAPK3,PRKCI,CHRM1,HRH2,CHRM5,CHRM2,CHRNA7,CHRM4,NR4A1
Cellular response to organonitrogen compound	1.11E-06	APEX1,CHRM3,MAPK3,PRKCI,CHRM1,HRH2,CHRM5,CHRM2,CHRM4,NR4A1
Cellular response to chemical stimulus	2.10E-06	PPP5C,APEX1,CHRM3,CYP2C9,MAPK3,PRKCI,CHRM1,OXSR1,CYP2B6,CDK19,HRH2,CHRM5,CHRM2,ESR1,CHRNA7,CHRM4,NR4A1
Response to organic cyclic compound	3.87E-06	PPP5C,APEX1,CHRM3,MAPK3,CHRM1,HRH2,CHRM5,CHRM2,ESR1,CHRM4,NR4A1
Protein phosphorylation	5.65E-06	PPP5C,MAPK6,MAPK3,CAMK4,PRKCI,OXSR1,CDK19,ZAK,TSSK1B,CIT,MAP2K7
Signal transduction	7.28E-06	PPP5C,PARP2,CHRM3,MAPK6,MAPK3,CAMK4,PRKCI,CHRM1,OXSR1,ZAK,HRH2,CHRM5,TSSK1B,CIT,MAP2K7,CHRM2,ESR1,CHRNA7,CHRM4,NR4A1
Response to chemical	9.39E-06	PPP5C,APEX1,CHRM3,CYP2C9,MAPK3,PRKCI,CHRM1,OXSR1,CYP2B6,CDK19,HRH2,CHRM5,MAP2K7,CHRM2,ESR1,CHRNA7,CHRM4,NR4A1,ABCB1
**C1**		
G protein-coupled receptor signaling pathway, coupled to cyclic nucleotide second messenger	1.16E-10	HTR2B,ADRA2A,HTR5A,MTNR1A,DRD5,ADRA1B,HRH2,ADRA1D,ADRA2C,ADRA2B
Vascular process in circulatory system	3.00E-10	HTR2B,ADRA2A,DRD5,ADRA1B,HRH2,ADRA1D,ADRA2C,ABCB1,ADRA2B
Adenylate cyclase-activating adrenergic receptor signaling pathway	4.05E-10	ADRA2A,DRD5,ADRA1B,ADRA1D,ADRA2C,ADRA2B
Regulation of blood vessel diameter	1.20E-09	HTR2B,ADRA2A,DRD5,ADRA1B,HRH2,ADRA1D,ADRA2C,ADRA2B
Cyclic-nucleotide-mediated signaling	4.61E-09	HTR2B,ADRA2A,HTR5A,DRD5,ADRA1B,ADRA1D,ADRA2C,ADRA2B
Adenylate cyclase-modulating g protein-coupled receptor signaling pathway	2.64E-08	ADRA2A,HTR5A,MTNR1A,DRD5,ADRA1B,ADRA1D,ADRA2C,ADRA2B
Regulation of vasoconstriction	3.32E-08	ADRA2A,ADRA1B,HRH2,ADRA1D,ADRA2C,ADRA2B
Exogenous drug catabolic process	4.53E-08	CYP2C9,CYP2B6,CYP3A4,CYP2D6,CYP2C19
cAMP-mediated signaling	8.96E-08	ADRA2A,HTR5A,DRD5,ADRA1B,ADRA1D,ADRA2C,ADRA2B
Monoterpenoid metabolic process	1.22E-07	CYP2C9,CYP3A4,CYP2D6,CYP2C19
Response to drug	3.35E-06	HTR2B,CYP2C9,CYP2B6,CYP3A4,CYP2D6,CYP2C19,ABCB1
Cell-cell signaling	1.10E-05	HTR2B,ADRA2A,HTR5A,DRD5,ADRA1B,HRH2,ADRA1D,ADRA2C,CHRNA7,ADRA2B
Negative regulation of epinephrine secretion	1.73E-05	ADRA2A,ADRA2C,ADRA2B
Phospholipase c-activating g protein-coupled receptor signaling pathway	2.11E-05	HTR2B,ADRA2A,DRD5,ADRA1B,ADRA1D
Positive regulation of vasoconstriction	2.11E-05	ADRA1B,HRH2,ADRA1D,ADRA2C
**APO**		
G protein-coupled receptor signaling pathway, coupled to cyclic nucleotide second messenger	3.77E-14	DRD4,HTR2B,ADRA2A,HTR5A,HTR6,DRD5,ADRA1B,HTR1A,HTR7,HRH2,DRD1,ADRA2C,OPRM1,ADRA2B
Cellular response to dopamine	1.30E-12	DRD4,HTR2B,HTR5A,HTR6,DRD5,HTR1A,HTR7,HRH2,DRD1,OPRM1
Vascular process in circulatory system	1.77E-11	HTR2B,ADRA2A,DRD5,ADRA1B,HTR1A,HTR7,HRH2,DRD1,ADRA2C,ABCB1,ADRA2B
Regulation of blood vessel diameter	5.17E-11	HTR2B,ADRA2A,DRD5,ADRA1B,HTR1A,HTR7,HRH2,DRD1,ADRA2C,ADRA2B
Chemical synaptic transmission	2.03E-09	DRD4,HTR2B,HTR5A,HTR6,DRD5,HTR1A,HTR7,HRH2,DRD1,OPRM1,CHRNA7,PIP5K1C
Adenylate cyclase-modulating g protein-coupled receptor signaling pathway	2.72E-09	DRD4,ADRA2A,HTR5A,DRD5,ADRA1B,HTR1A,DRD1,ADRA2C,OPRM1,ADRA2B
Adenylate cyclase-activating adrenergic receptor signaling pathway	3.24E-09	ADRA2A,DRD5,ADRA1B,DRD1,ADRA2C,ADRA2B
Cell-cell signaling	4.44E-09	DRD4,HTR2B,ADRA2A,HTR5A,HTR6,DRD5,ADRA1B,HTR1A,HTR7,HRH2,DRD1,ADRA2C,OPRM1,CHRNA7,PIP5K1C,ADRA2B
Cellular response to chemical stimulus	7.80E-09	DRD4,APEX1,HTR2B,CYP2C9,SIGMAR1,ADRA2A,HTR5A,HTR6,DRD5,OXSR1,HTR1A,CYP2B6,CYP3A4,HTR7,PTPN22,CYP2D6,CYP2C19,HRH2,DRD1,OPRM1,CHRNA7,PIP5K1C
Cyclic-nucleotide-mediated signaling	8.67E-09	HTR2B,ADRA2A,HTR5A,DRD5,ADRA1B,DRD1,ADRA2C,OPRM1,ADRA2B
Cellular response to nitrogen compound	8.67E-09	DRD4,APEX1,HTR2B,HTR5A,HTR6,DRD5,HTR1A,HTR7,PTPN22,HRH2,DRD1,OPRM1,CHRNA7
G protein-coupled receptor signaling pathway	1.40E-08	DRD4,HTR2B,SIGMAR1,ADRA2A,HTR5A,HTR6,DRD5,ADRA1B,HTR1A,GRK1,HTR7,HRH2,DRD1,ADRA2C,OPRM1,ADRA2B
Cellular response to stimulus	1.40E-08	DRD4,APEX1,MAP3K10,HTR2B,CYP2C9,SIGMAR1,ADRA2A,HTR5A,HTR6,DRD5,ADRA1B,OXSR1,HTR1A,CYP2B6,GRK1,CYP3A4,HTR7,PKN1,PTPN22,CYP2D6,CYP2C19,HRH2,DRD1,STK32A,ADRA2C,OPRM1,CHRNA7,PIP5K1C,ADRA2B
Response to stimulus	1.41E-08	DRD4,APEX1,MAP3K10,HTR2B,CYP2C9,SIGMAR1,ADRA2A,HTR5A,HTR6,DRD5,ADRA1B,OXSR1,HTR1A,PLEC,CYP2B6,GRK1,CYP3A4,HTR7,PKN1,PTPN22,CYP2D6,CYP2C19,HRH2,DRD1,STK32A,ADRA2C,OPRM1,CHRNA7,PIP5K1C,ABCB1,ADRA2B
Cellular response to organonitrogen compound	4.92E-08	DRD4,APEX1,HTR2B,HTR5A,HTR6,DRD5,HTR1A,HTR7,PTPN22,HRH2,DRD1,OPRM1

Suppl. Table 2

Term Description	FDR q-value	Matching Proteins
C1		
G protein-coupled receptor signaling pathway, coupled to cyclic nucleotide second messenger	5.93E-10	MTNR1B,MTNR1A,DRD5,HTR1A,PTGDR2,HTR7,DRD2,AGTR2,DRD3,DRD1,HTR2A
Chemical synaptic transmission	5.93E-10	MTNR1B,DRD5,HTR1A,HTR7,DRD2,SLC22A2,TH,DRD3,DRD1,HCRTR1,CHRNA2,HTR2A,HCRTR2
Cellular response to dopamine	1.87E-07	DRD5,HTR1A,HTR7,DRD2,DRD3,DRD1,HTR2A
Synaptic transmission, dopaminergic	3.99E-07	DRD5,DRD2,TH,DRD3,DRD1
Vascular process in circulatory system	6.70E-07	MTNR1B,DRD5,HTR1A,HTR7,SLC22A2,AGTR2,DRD1,HTR2A
Circulatory system process	7.61E-07	MTNR1B,DRD5,HTR1A,HTR7,DRD2,SLC22A2,AGTR2,DRD3,DRD1,HTR2A
Cell-cell signaling	7.61E-07	MTNR1B,DRD5,HTR1A,HTR7,DRD2,SLC22A2,AGTR2,TH,DRD3,DRD1,HCRTR1,CHRNA2,HTR2A,HCRTR2
Behaviour	7.94E-07	MTNR1A,DRD5,HTR1A,DRD2,AGTR2,TH,DRD3,DRD1,HCRTR1,HTR2A,HCRTR2
Dopamine metabolic process	1.67E-06	DRD2,AGTR2,TH,DRD3,DRD1
Regulation of biological quality	2.42E-06	MTNR1B,SLC22A3,PRELID1,DRD5,HTR1A,F3,HTR7,TUBB,DRD2,PARP1,SLC22A2,AGTR2,TH,DRD3,DRD1,PLAA,PARP3,FKBP1A,HCRTR1,CHRNA2,HTR2A,HCRTR2
Regulation of blood vessel diameter	2.42E-06	MTNR1B,DRD5,HTR1A,HTR7,AGTR2,DRD1,HTR2A
Phospholipase c-activating dopamine receptor signaling pathway	2.89E-06	DRD5,DRD2,DRD3,DRD1
Response to amphetamine	2.98E-06	DRD5,DRD2,TH,DRD3,DRD1
Phenol-containing compound metabolic process	4.43E-06	HTR1A,DRD2,AGTR2,TH,DRD3,DRD1
Blood circulation	5.69E-06	MTNR1B,DRD5,HTR1A,HTR7,DRD2,AGTR2,DRD3,DRD1,HTR2A
APO		
Cellular response to dopamine	4.38E-09	DRD4,DRD5,HTR1A,DRD2,DRD3,DRD1
Response to amphetamine	6.26E-09	DRD4,DRD5,DRD2,DRD3,DRD1
Regulation of dopamine uptake involved in synaptic transmission	1.78E-08	DRD4,DRD2,DRD3,DRD1
Response to cocaine	2.15E-08	DRD4,DRD5,DRD2,DRD3,DRD1
Phospholipase c-activating dopamine receptor signaling pathway	2.15E-08	DRD5,DRD2,DRD3,DRD1
Behavioural response to cocaine	4.37E-08	DRD4,DRD2,DRD3,DRD1
Adenylate cyclase-modulating g protein-coupled receptor signaling pathway	1.96E-07	DRD4,DRD5,HTR1A,DRD2,DRD3,DRD1
Phenol-containing compound metabolic process	2.22E-07	DRD4,HTR1A,DRD2,DRD3,DRD1
Adenylate cyclase-activating adrenergic receptor signaling pathway	2.27E-07	DRD5,DRD2,DRD3,DRD1
Dopamine metabolic process	5.37E-07	DRD4,DRD2,DRD3,DRD1
Cellular response to organonitrogen compound	5.45E-07	DRD4,DRD5,HTR1A,DRD2,PARP1,DRD3,DRD1
Adenylate cyclase-inhibiting dopamine receptor signaling pathway	2.28E-06	DRD4,DRD2,DRD3
Chemical synaptic transmission	4.48E-06	DRD4,DRD5,HTR1A,DRD2,DRD3,DRD1
Negative regulation of synaptic transmission	7.80E-06	DRD5,DRD2,DRD3,DRD1
Response to organic cyclic compound	7.86E-06	DRD4,DRD5,HTR1A,DRD2,PARP1,DRD3,DRD1

Suppl. Table 3

Gene Symbol	Forward (F) and Reverse (R) Primers Sequences (5'- 3')
GAPDH	F - GGAGTCAACGGATTTGGTCGTA<br>R - GGCAACAATATCCACTTTACCA
PROM1 (CD133)	F - ACCCAACATCATCCCTGTTCTT<br>R - AGCTCTTCAAGGTGCTGTTCATG
NES	F - AGCCCTGACCACTCCAGTTTAG<br>R - CCCTCTATGGCTGTTTCTTTCTCT
OCT4	F - AGTGCCCGAAACCCACACT<br>R - CTTCTGGCGCCGGTTACA
CD44	F - ATAATTGCCGCTTTGCAGGTGTATT<br>R - ATAATGGCAAGGTGCTATTGAAAGCCT
BAX	F - CGACTGATGTCCCTGTCTCC<br>R - CTCCCGCCACAAAGATGGT
BECN1	F - GCTCCCGAGGTGAAGAGCAT<br>R - TGTGGTAAGTAATGGAGCTGTGA
BCL2L11 (BIM)	F - CACTATCTCAGTGCAATGGCTTCC<br>R - ATTCGTGGGTGGTCTTCGG
CDKN1A (p21)	F - CTGCCCAAGCTCTACCTTCC<br>R - CAGGTCCACATGGTCTTCCT
CCNB1	F - ACCTGTGTCAGGCTTTCTCTG<br>R - TGGTCTGACTGCTTGCTCTTC
ABCB1	F - GTCTGGACAAGCACTGAAA<br>R - AACAACGGTTCGGAAGTTT
CYP2B6	F - AGACGCCTTCAATCCTGACC<br>R - CCTTCACCAAGACAAATCCGC
CYP2C9	F - TGGATGAAGGTGGCAATTTT<br>R - GGGCTTCTCCCACACAAAT
DRD1	F - CTTCCTCAACGTTTCGGAGCC<br>R - AGCTCTCCAAACGCCTTGCCTT
DRD5	F - TCATCTATGCCTTCAACGCCGACT<br>R - AGCTGCGATTTCCTTGTGGAAGAC

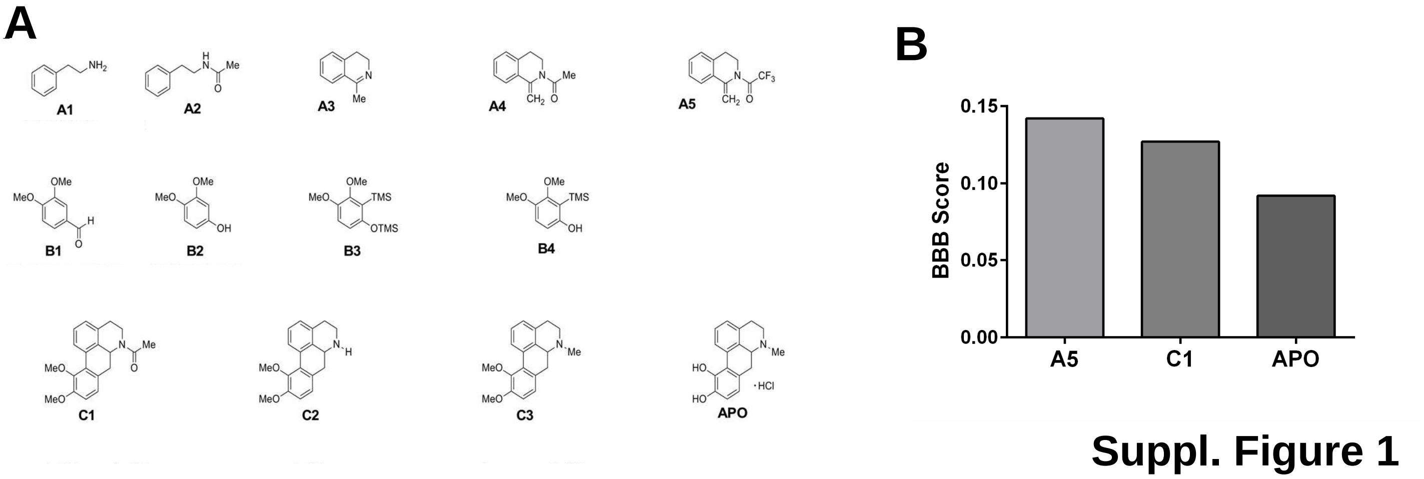

Suppl. Figure 1

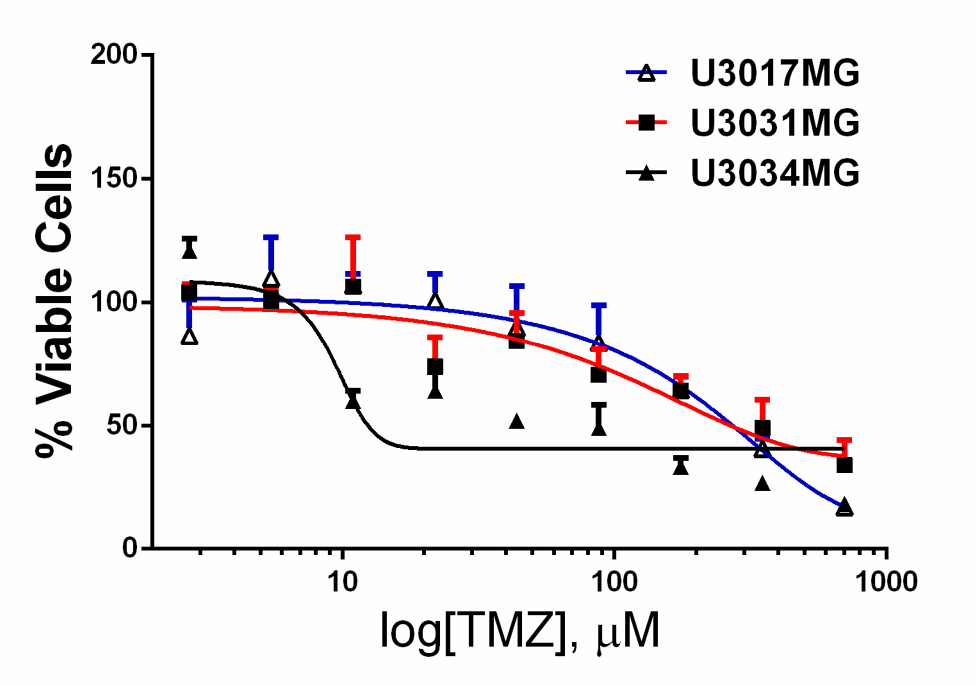

Suppl. Figure 2

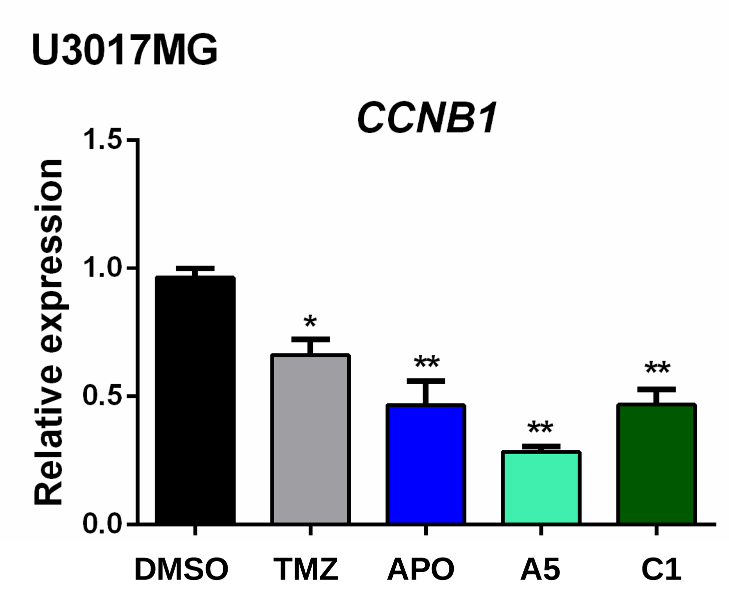

Suppl. Figure 3

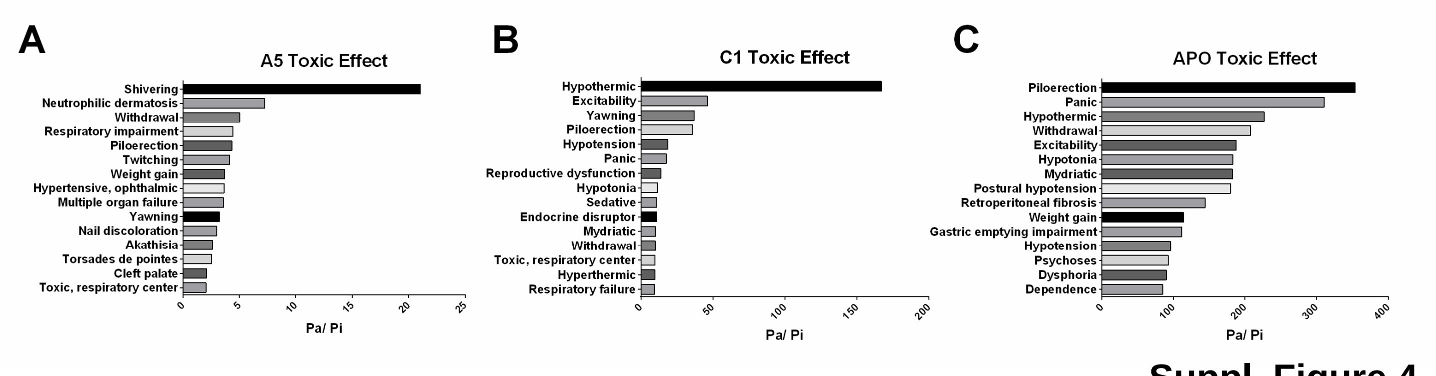

Suppl. Figure 4

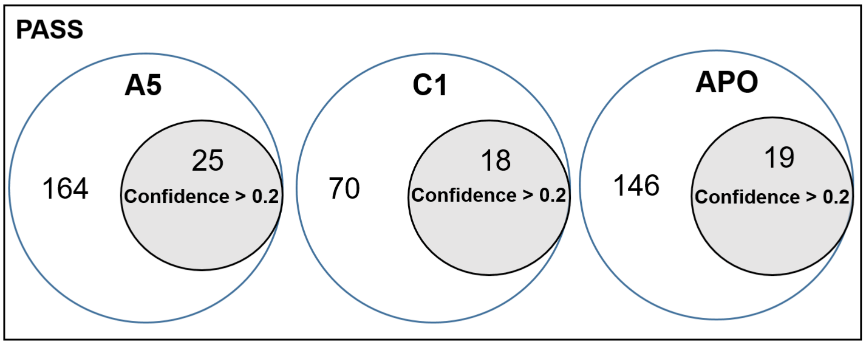

Suppl. Figure 5

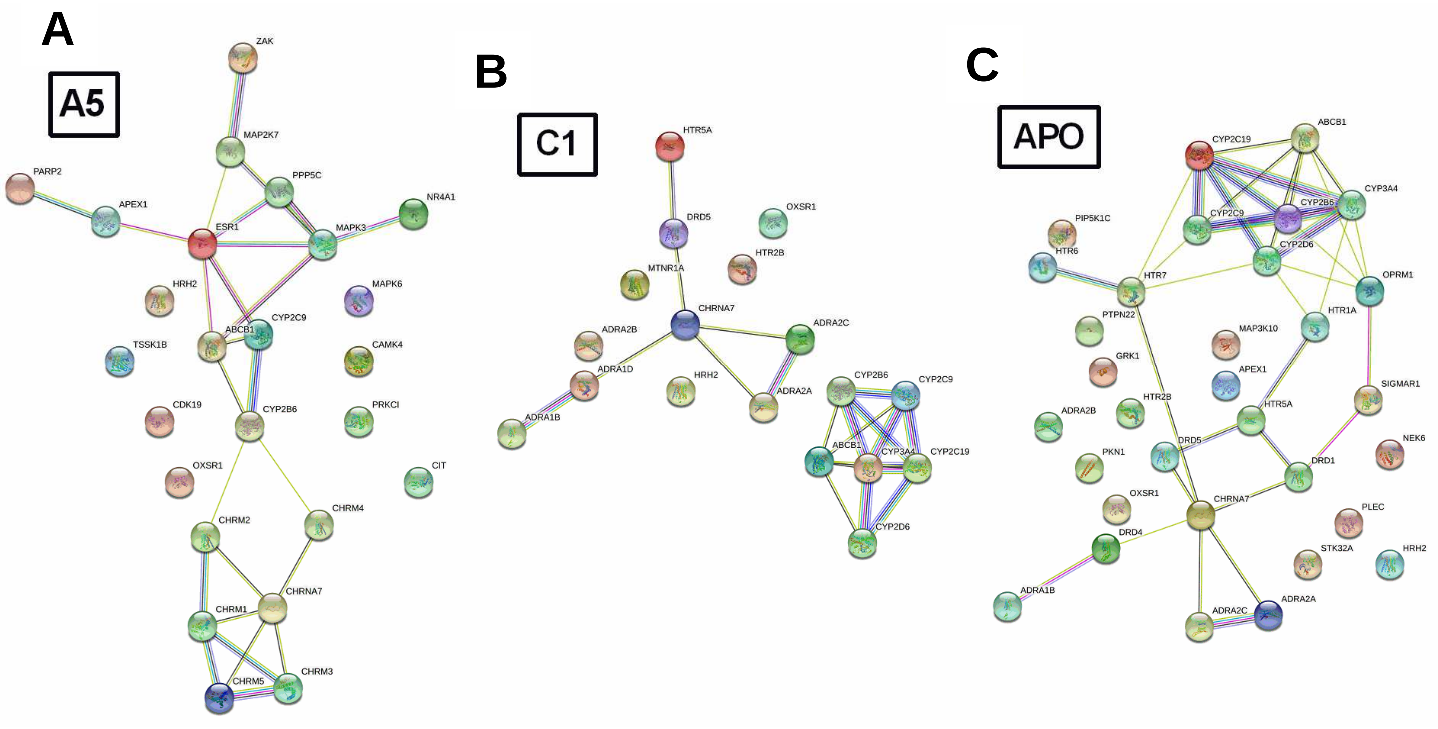

Suppl. Figure 6

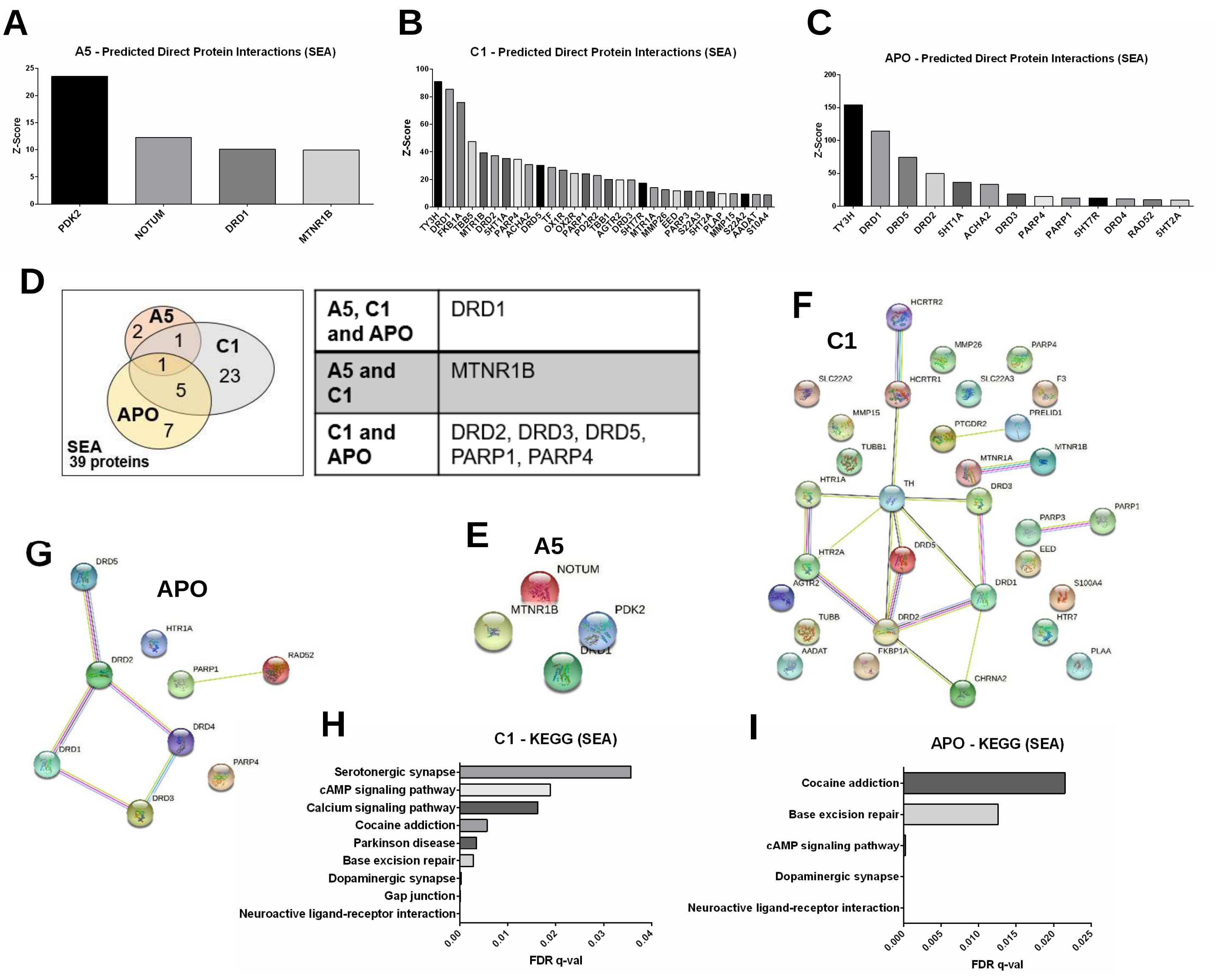

Suppl. Figure 7

A

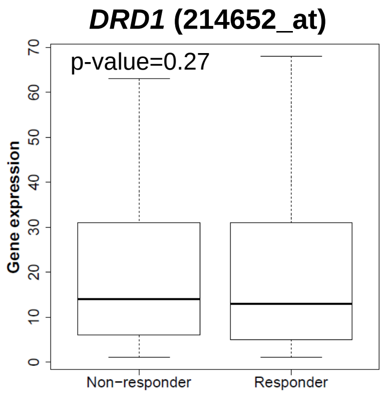

TMZ response	N of patients	Mean	SD	Median	Minimum	Maximum
**Responder**	165	21	28	13	1	264
**Non-responder**	154	27	41	14	1	314

B

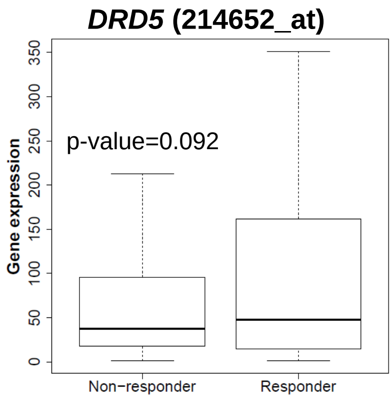

TMZ response	N of patients	Mean	SD	Median	Minimum	Maximum
**Responder**	165	91	92	48	1	351
**Non-responder**	154	79	97	38	1	646

C

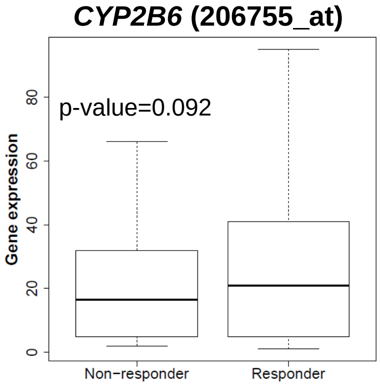

TMZ response	N of patients	Mean	SD	Median	Minimum	Maximum
**Responder**	165	34	40	21	1	202
**Non-responder**	154	24	28	16	2	161

D

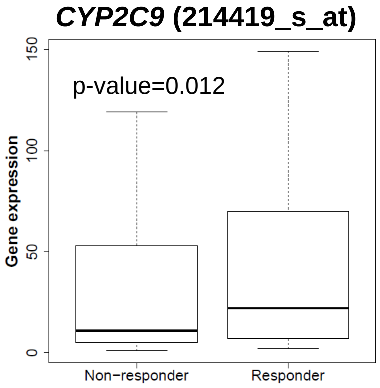

TMZ response	N of patients	Mean	SD	Median	Minimum	Maximum
**Responder**	165	41	42	22	2	165
**Non-responder**	154	36	54	11	1	482

Suppl. Figure 8

CYP2C9

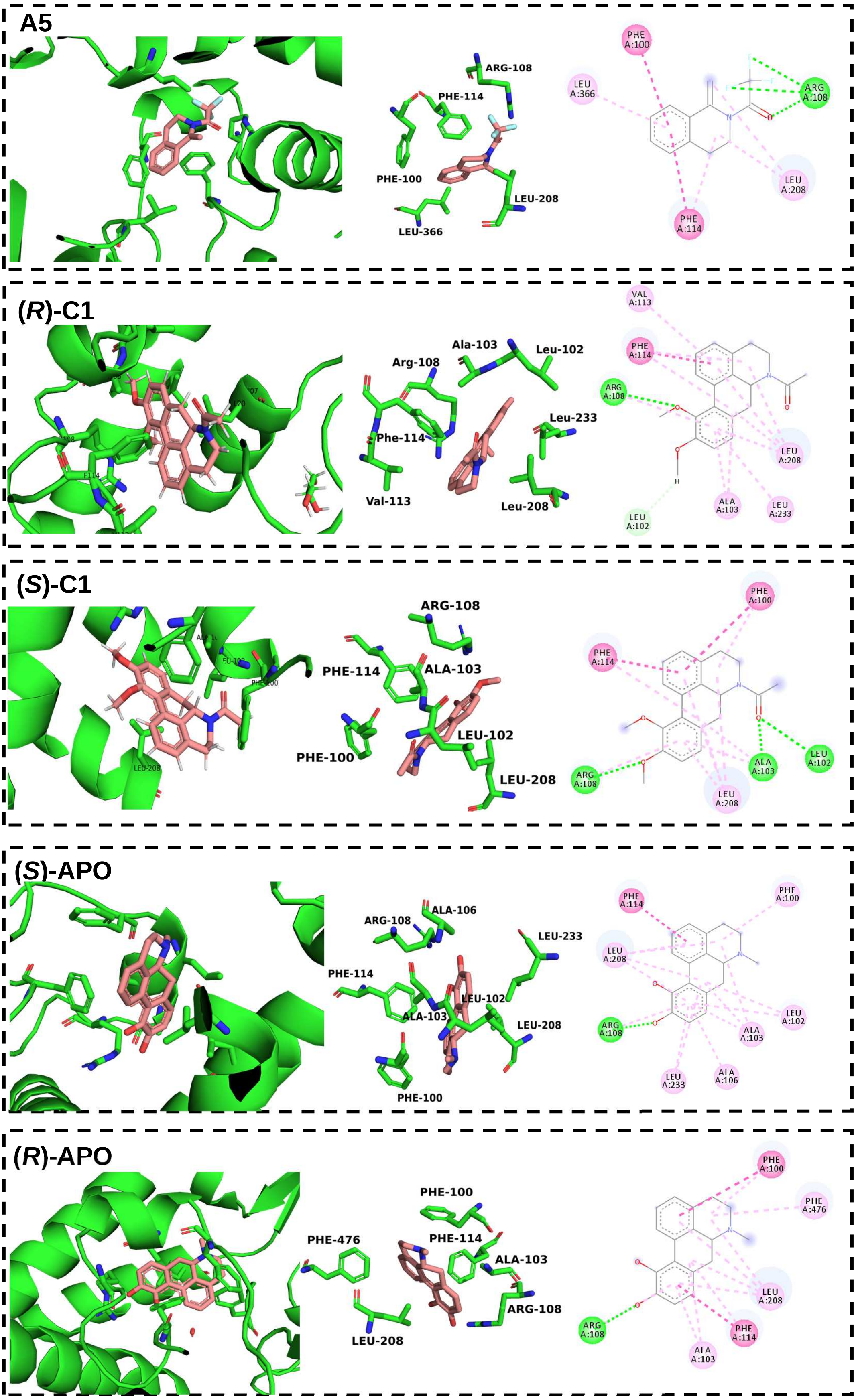

Interactions

- Conventional Hydrogen Bond
- Carbon Hydrogen Bond
- Pi-Stacked
- Alkyl

ΔG Binding Energy (kcal/mol)

A5 = -25.69
(*R*)-C1 = -36.62
(*S*)-C1 = -38.40
(*R*)-APO = -35.45
(*S*)-APO = -28.51

Suppl. Figure 9

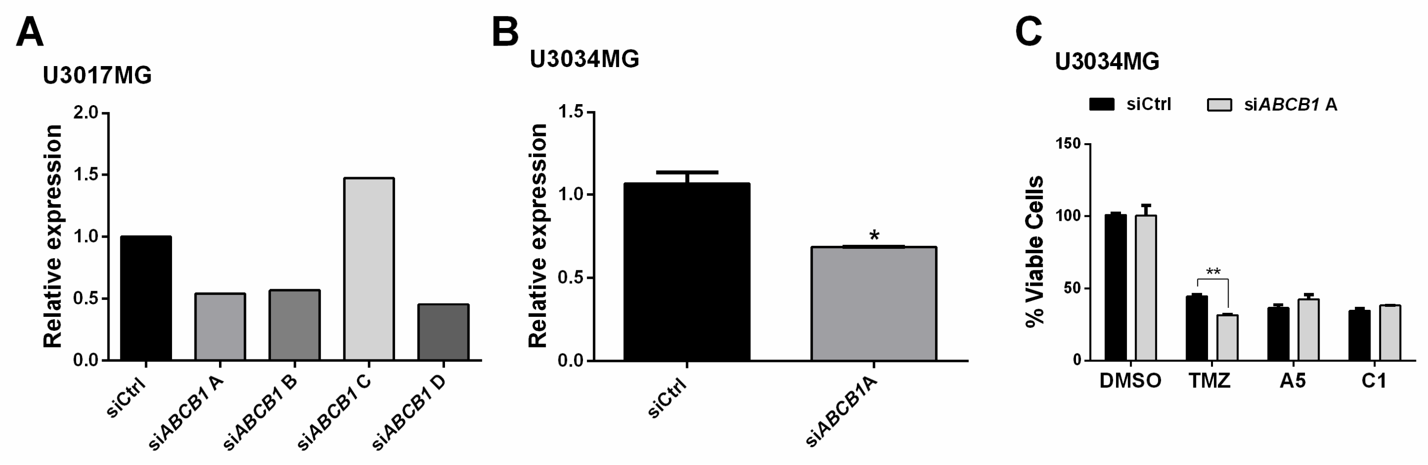

Suppl. Figure 10

Unprocessed immunoblots

(to Fig. 2b)

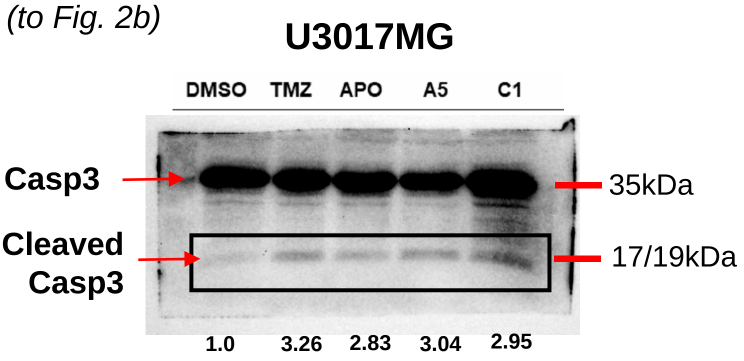

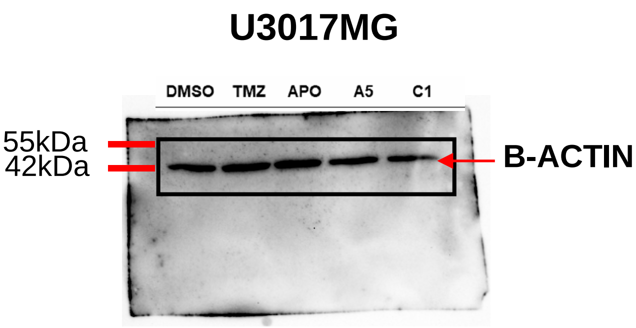

(to Fig. 3c)

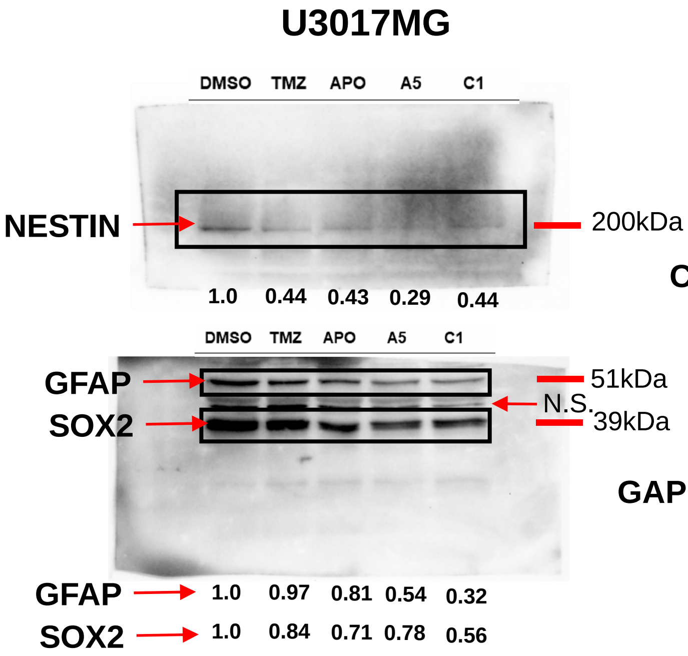

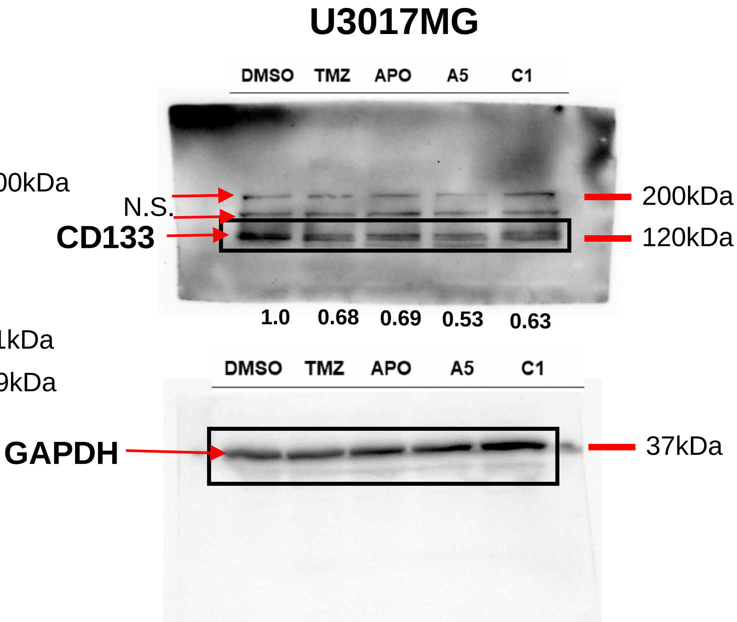

(to Fig. 6f)

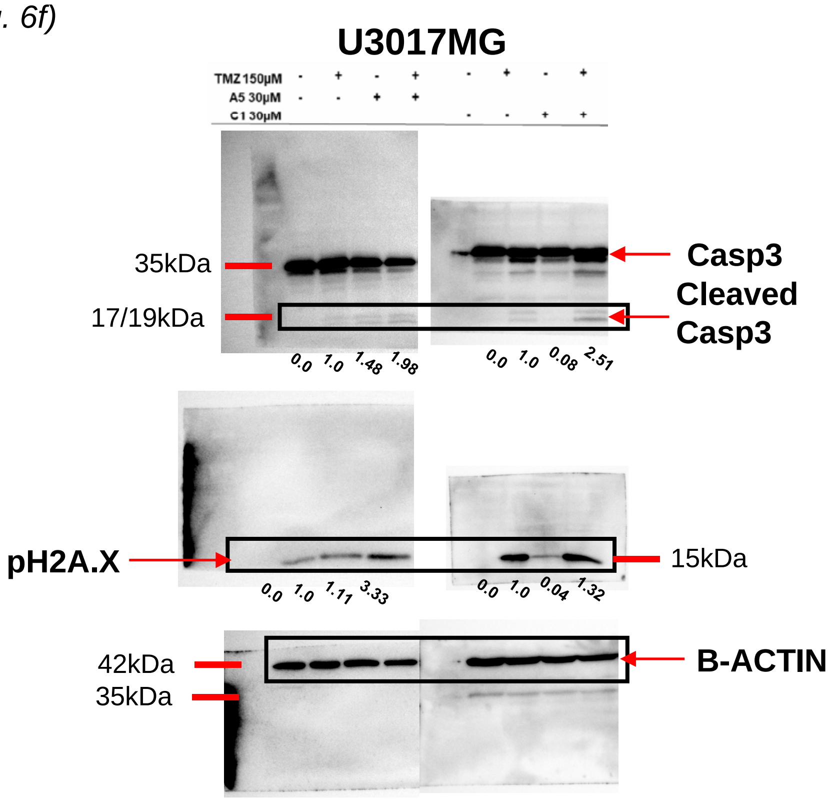

Suppl. Figure 11

A

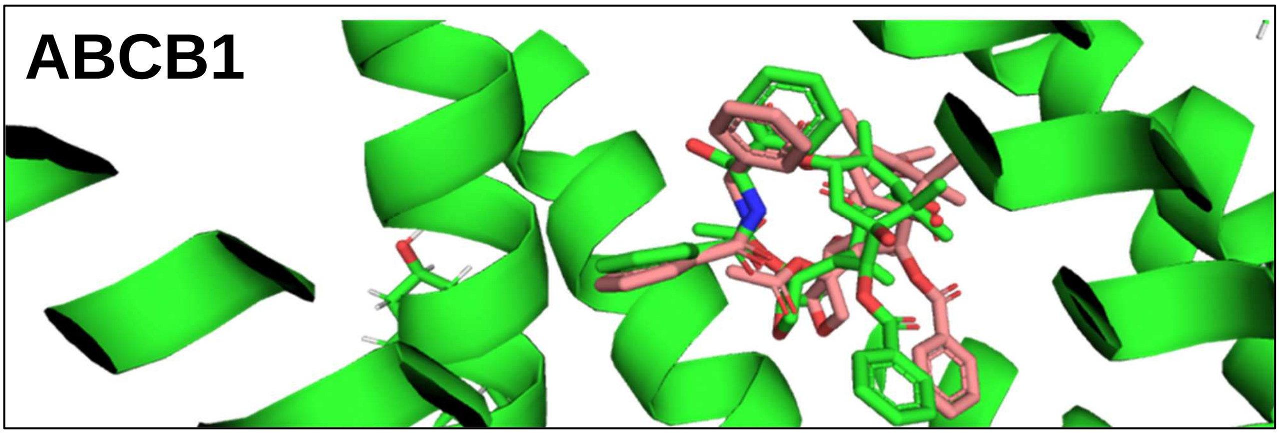

B

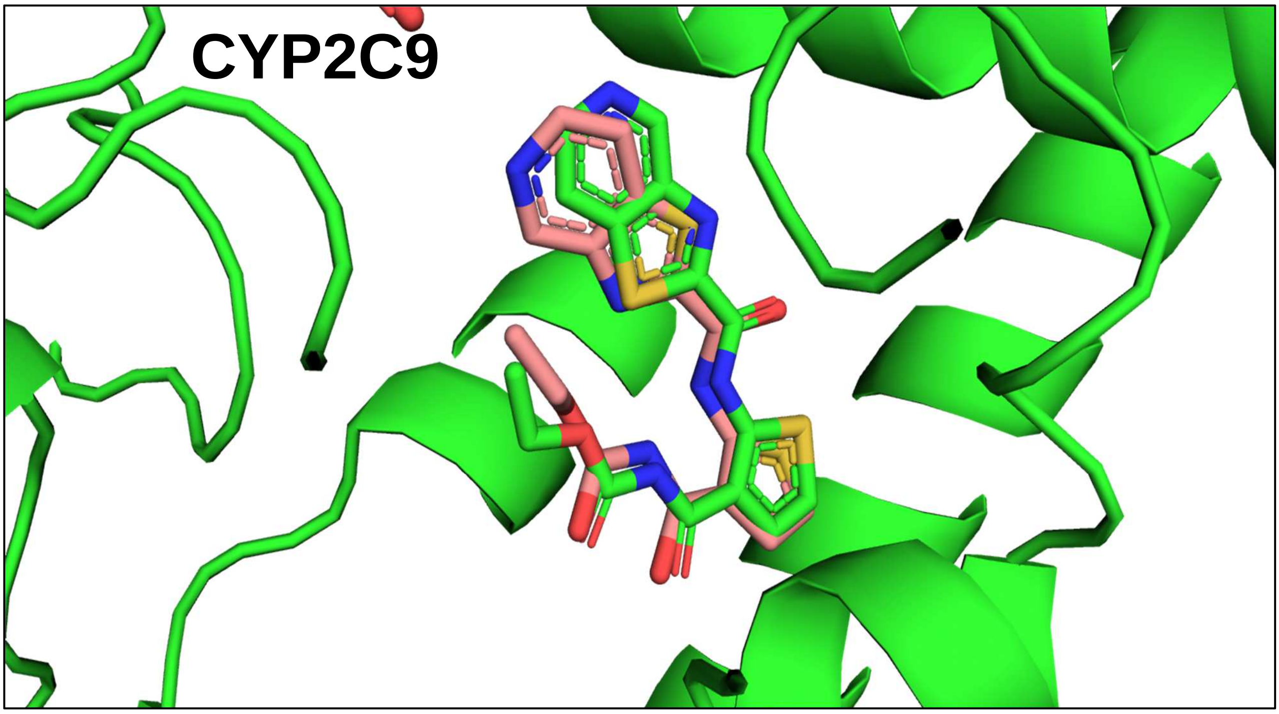

Suppl. Figure 12
